# *N*-Hydroxy pipecolic acid methyl ester is involved in Arabidopsis immunity

**DOI:** 10.1093/jxb/erac422

**Published:** 2022-10-19

**Authors:** Lennart Mohnike, Weijie Huang, Brigitte Worbs, Kirstin Feussner, Yuelin Zhang, Ivo Feussner

**Affiliations:** University of Goettingen, Albrecht-von-Haller-Institute for Plant Sciences, Department of Plant Biochemistry, D-37077 Goettingen, Germany; University of British Columbia, Department of Botany, V6T 1Z4 Vancouver (BC), Canada; University of Goettingen, Institute for Organic and Biomolecular Chemistry, Department of Organic Chemistry, D-37077 Goettingen, Germany; University of Goettingen, Albrecht-von-Haller-Institute for Plant Sciences, Department of Plant Biochemistry, D-37077 Goettingen, Germany; University of Goettingen, Goettingen Center for Molecular Biosciences (GZMB), Service Unit for Metabolomics and Lipidomics, D-37077 Goettingen, Germany; University of British Columbia, Department of Botany, V6T 1Z4 Vancouver (BC), Canada; University of Goettingen, Albrecht-von-Haller-Institute for Plant Sciences, Department of Plant Biochemistry, D-37077 Goettingen, Germany; University of Goettingen, Goettingen Center for Molecular Biosciences (GZMB), Department of Plant Biochemistry, D-37077 Goettingen, Germany; Universitat Jaume I, Spain

**Keywords:** *Arabidopsis thaliana*, *N*-hydroxy pipecolic acid methyl ester, infection metabolism, non-targeted metabolomics, plant immunity, salicylic acid, UV stress

## Abstract

The biosynthesis of *N*-hydroxy pipecolic acid (NHP) has been intensively studied, though knowledge on its metabolic turnover is still scarce. To close this gap, we discovered three novel metabolites via metabolite fingerprinting in *Arabidopsis thaliana* leaves after *Pseudomonas* infection and UV-C treatment. Exact mass information and fragmentation by tandem mass spectrometry (MS/MS) suggest a methylated derivative of NHP (MeNHP), an NHP-*O*Glc-hexosyl conjugate (NHP-*O*Glc-Hex), and an additional NHP-*O*Glc-derivative. All three compounds were formed in wild-type leaves but were not present in the NHP-deficient mutant *fmo1-1*. The identification of these novel NHP-based molecules was possible by a dual-infiltration experiment using a mixture of authentic NHP and D_9_-NHP standards for leaf infiltration followed by UV-C treatment. Interestingly, the signal intensity of MeNHP and other NHP-derived metabolites increased in *ugt76b1-1* mutant plants. For MeNHP, we unequivocally determined the site of methylation at the carboxylic acid moiety. MeNHP application by leaf infiltration leads to the detection of a MeNHP-*O*Glc as well as NHP, suggesting MeNHP hydrolysis to NHP. This is in line with the observation that MeNHP infiltration is able to rescue the *fmo1-1* susceptible phenotype against *Hyaloperonospora arabidopsidis* Noco 2. Together, these data suggest MeNHP as an additional storage or transport form of NHP.

## Teaser

We identify *N*-hydroxy pipecolic acid (NHP) metabolites including its methyl ester and complex glycosides. The application of NHP methyl ester rescues the disease phenotype of an NHP biosynthesis-deficient mutant.

## Introduction

Plants experience reduced growth or early senescence if they are unable to maintain a balance between growth and defense (von Saint Paul *et al.*, 2011; Zhang *et al.*, 2017). Their immune system depends on a tightly regulated and highly dynamic balance of activation and inactivation (Karasov *et al.*, 2017; Zeier, 2021). Salicylic acid (SA) and *N*-hydroxy pipecolic acid (NHP) are two key molecules which act in concert in the defense response against (hemi-)biotrophic pathogens (Fu and Dong, 2013; Hartmann and Zeier, 2019).

**Fig. 1.**
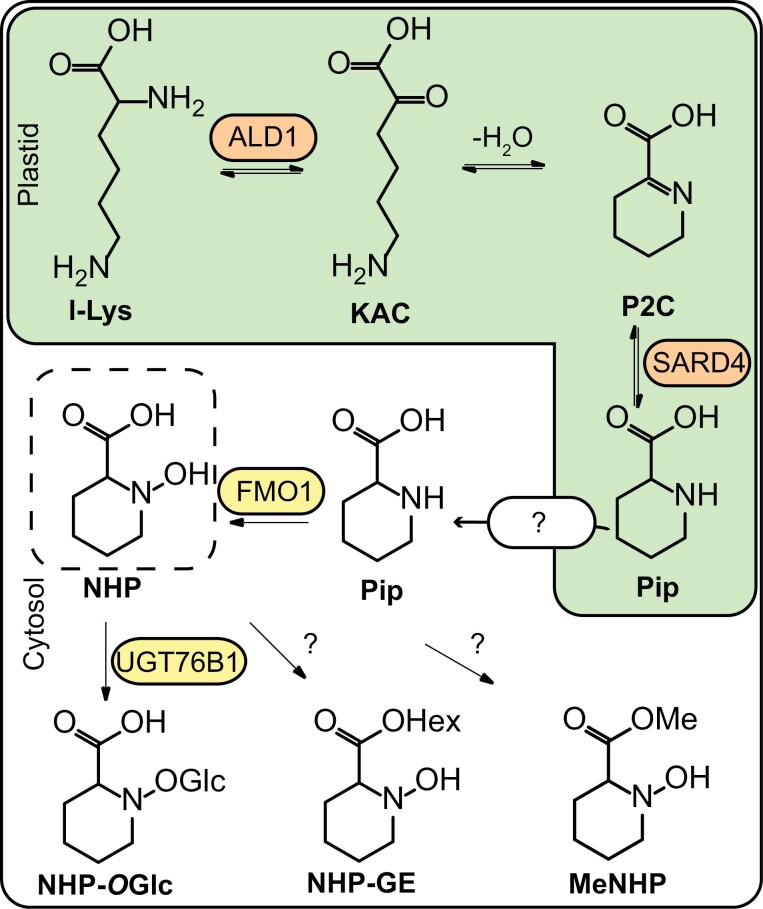
Biosynthesis route of NHP metabolites in Arabidopsis. In the plastid, l-lysine (l-Lys) is converted by AGD2-LIKE DEFENSE RESPONSE PROTEIN 1 (ALD1) to epsilon-amino-alpha-keto caproic acid (KAC). Via spontaneous cyclization of KAC under water loss, piperidein-2-carboxylic acid (P2C) is formed. A reductase capable of reducing P2C to pipecolic acid (Pip) is SYSTEMIC ACQUIRED RESISTANCE DEFICIENT 4 (SARD4). How pipecolic acid is exported from the chloroplast is still elusive. The FLAVIN-DEPENDENT MONOOXYGENASE 1 (FMO1) catalyzes N-hydroxylation of Pip, resulting in N-hydroxy pipecolic acid (NHP) (Navarova *et al*., 2012; Chen *et al*., 2018; Ding *et al*., 2016; Hartmann *et al*., 2018). NHP was shown to be glycosylated by UGT76B1 to NHP-O-glycoside (NHP-O-Glc). Furthermore, NHP glycoside ester (NHP-GE) was described, but the respective enzyme is not known (Hartmann and Zeier, 2018; Bauer *et al*., 2021). Methylation of NHP to NHP methyl ester (MeNHP) is shown as an additional mechanism of NHP turnover in planta in this study.

In the Brassicaceae model organism *Arabidopsis thaliana*, SA and NHP are synthesized upon pathogen infection and UV-C treatment (Mohnike *et al.*, 2021). Roughly 90% of SA derives from chorismic acid, which is converted via the ISOCHORISMIC ACID SYNTHASE 1 (ICS1) pathway. This pathway features the enzymes AvrPphB SUSCEPTIBLE 3 (PBS3) and ENHANCED PSEUDOMONAS SUSCEPTIBILITY 1 (EPS1) to synthesize SA (Wildermuth *et al.*, 2001; Rekhter *et al.*, 2019; Torrens-Spence *et al.*, 2019). NHP derives from l-lysine via the enzymatic route of AGD2-LIKE DEFENSE RESPONSE PROTEIN 1 (ALD1), SYSTEMIC ACQUIRED RESISTANCE DEFICIENT 4 (SARD4), and FLAVINE-DEPENDENT MONOOXYGENASE 1 (FMO1) (Fig. 1). Both molecules orchestrate defense signaling including the activation of defense gene expression and danger signal amplification (Navarova *et al.*, 2012; Ding *et al.*, 2016; Chen *et al.*, 2018; Hartmann *et al.*, 2018). In consequence, distant leaves are primed for robust defense against secondary stressors. This process is termed systemic acquired resistance (SAR) (Chen *et al.*, 2018; Hartmann *et al.*, 2018). That SA plays a crucial role in the establishment of SAR has been known for years (Gaffney *et al.*, 1993). Recently, NHP has been described as a mobile inducer of SAR (Chen *et al.*, 2018; Hartmann *et al.*, 2018). NHP confers broad-spectrum disease resistance against pathogens such as *Pseudomonas* and oomycetes, for instance *Hyaloperonospora arabidopsidis*, in Arabidopsis (Chen *et al.*, 2018; Hartmann *et al.*, 2018). Moreover, NHP was shown to be synthesized in various mono- and dicotyledonous plants in response to pathogens (Holmes *et al.*, 2019; Schnake *et al.*, 2020). Similar to Arabidopsis, NHP pre-treatment of, for example, *Cucumis sativus*, *Nicotiana tabacum*, and *Brachypodium distachyon* resulted in enhanced disease resistance against the respective pathogens (Schnake *et al.*, 2020). In *C. sativus*, NHP accumulated in petiole exudates from *Pseudomonas*-inoculated leaves and in exudates from distant leaves, which hints at vascular mobility (Schnake *et al.*, 2020). To mimic metabolic changes after pathogen infection in Arabidopsis, such as SA and NHP accumulation, in an easy and highly reproducible way, UV-C treatment can be used (Mohnike *et al.*, 2021).

One way to regulate cellular concentrations of bioactive compounds is by metabolic turnover. For instance, UDP-dependent glycosyltransferases (UGTs) have been shown to form glucosides and glucoside esters of phytohormones (Dean and Delaney, 2008; Noutoshi *et al.*, 2012; George Thompson *et al.*, 2017; Maksym *et al.*, 2018; Haroth *et al.*, 2019). Further mechanisms of turnover can be hydroxylation or methylation of the active compound as known for SA (Chen *et al.*, 2003; Park *et al.*, 2007; Zhang *et al.*, 2013; Zhang *et al.*, 2017). For NHP, so far two products of turnover have been described. NHP glucosylation was identified in several independent studies resulting in the formation of NHP-*O*Glc (Bauer *et al.*, 2021; Cai *et al.*, 2021; Holmes *et al.*, 2021; Mohnike *et al.*, 2021). UGT76B1 was shown to be responsible for the formation of NHP-*O*Glc *in vivo*. The *ugt76b1* mutant plants exhibited enhanced resistance to *Pseudomonas syringae* and *H. arabidopsidis* Noco 2 in an *FMO1*-dependent manner (Bauer *et al.*, 2021; Mohnike *et al.*, 2021). Via heterologous expression of NHP biosynthetic genes with and without expression of UGT76B1, it was shown that glycosylation by UGT76B1 inactivates NHP (Holmes *et al.*, 2021). In addition, a second glycoside form, NHP-Glc ester (NHP-GE), has been proposed (Bauer *et al.*, 2021). Nevertheless, the identification of NHP metabolites may be incomplete. Other modifications, such as methylation or amino acid conjugation, have not yet been described for NHP.

Here we report infection and UV-C-dependent formation of methylated NHP (MeNHP) identified via ultra-high-performance LC high-resolution MS (UHPLC-HRMS) metabolome analysis. We confirm NHP methylation via D_9_-labeled NHP and determined carboxylic acid methylation via a comparison with a synthesized authentic MeNHP standard. Furthermore, we show that MeNHP is able to rescue the NHP-deficient phenotype of *fmo1-1* mutant plants and reduce oomycete spore growth on *A. thaliana*. In addition, we present a dual-infiltration experiment with a mixture of NHP and D_9_-NHP to identify and investigate novel metabolites of NHP in a non-targeted manner.

## Materials and methods

### Plant material and growth conditions

*A. thaliana* ecotype Col-0, *fmo1-1*, *ugt76b1-1*, *fmo1 ugt76b1* (CRISPR *ugt76b1-5* in *fmo1-1*), and *FMO1-3D* were used in this study (Mohnike *et al.*, 2021), together with *FMO1-3D ald1*. We obtained *nhpmt1-1* (SALK_053006) and *nhpmt1-2* (SALKseq_135601) mutant plants from the SALK Institute, Nottingham. Plants were grown on steam-sterilized soil under short days (8 h light/16 h dark) or long days (16 h light/8 h dark) for 4 and 6 weeks. The light intensity was 100–120 µmol m^–2^ s^–1^ and the relative humidity was 80%, unless specified otherwise. The light source was MASTER LED tubes HF 600 mm HO 8W840 T8 (PHILIPS AG, Amsterdam, the Netherlands).

### *Pseudomonas* infection and UV treatment

To induce defense metabolism, plants were treated with *Pseudomonas* or UV-C light. *Pseudomonas syringae* strain ES4326 (*P.s.m.*) were grown in LB medium with 25 µg of rifampicin overnight at 28 °C. The culture was pelleted, the medium was decanted, and the bacteria were suspended in 10 mM MgCl_2_. Bacteria were diluted to OD_600_=0.05 and infiltrated on the abaxial side of the leaf. As mock treatment control, 10 mM MgCl_2_ was infiltrated onto the leaf. Plants were incubated for 8, 24, or 48 h, as stated in the Results and figure legends. UV-C treatment was performed for 20 min in a PrettleTelstar sterile bench as described (Mohnike *et al.*, 2021). Plants were incubated for 24 h post-treatment if not stated otherwise.

### Chemical synthesis of MeNHP

MeNHP was synthesized from methyl pipecolinate hydrochloride after a modified procedure (Sousa *et al.*, 2016). All solvents [ethyl acetate (EtOAc), hexane, diethylether (Et_2_O), and dichloromethane (DCM) of technical grade unless stated otherwise] used for work-up procedures and flash column chromatography were distilled prior to use. Methanol (MeOH), triethylamine (Et_3_N), and acrylonitrile used in reactions, were practical grade (p.a.) and supplied by Fisher Scientific (Loughborough, UK) and Sigma Aldrich (Steinheim, Germany). Methylpipecolinate hydrochloride and *m*-chloroperoxybenzoic acid (*m*-CPBA) were from Sigma Aldrich and used as supplied. TLC was carried out on silica gel 60 F_254_ aluminum plates (Merck, Darmstadt, Germany). Substances were detected under UV at 254 nm and dipping into a ninhydrin solution (3% in EtOH) followed by heating. Flash column chromatography on silica was performed using Silica Gel 60 with a particle size of 0.063–0.2 mm (Machery-Nagel, Düren, Germany). The melting points were determined on a Stuart™ SMP10 melting point apparatus and were uncorrected. NMR spectra were recorded on an Advance Neo 400 spectrometer (Bruker, Billerica, MA, USA). Electrospray ionization (ESI)-MS and HRMS-ESI spectra were obtained with Bruker devices, maXis or MicrOTOF (Bremen, Germany).

Synthesis of *N*-(2-cyanoethyl)-methylpipecolinate was performed via the following procedure. Et_3_N (2.31 ml, 16.7 mmol) was added dropwise to the stirred suspension of methylpipecolinate hydrochloride (1.50 g, 8.35 mmol) in MeOH p.a. (8 ml) at 0 °C under argon. The resulting solution was stirred for 15 min at 0 °C. Then acrylonitrile (0.60 ml, 9.2 mmol) was added dropwise and, after 15 min at 0 °C, the reaction mixture was allowed to come to room temperature. Stirring was continued overnight. The solvent was removed by rotary evaporation. The residue was diluted with H_2_O (25 ml) and extracted with Et_2_O (3 × 25 ml). The organic phase was washed with saline (25 ml), dried over Na_2_SO_4_, filtered, and concentrated. The oily residue was purified by flash column chromatography (eluent, hexane:EtOAc, 3:1; R_f_, 0.65), affording a light yellow oil (1.02 g, 62%). Formation of *N*-(2-cyanoethyl)-methylpipecolinate was confirmed by (i) HRMS-ESI: calculated mass for [C_10_H_16_N_2_O_2_]^+^ (M+H^+^): 197.1285, found 197.1285 and for [C_10_H_16_N_2_O_2_]^+^ (M+Na^+^): 219.1104, found 219.1104 (Supplementary Fig. S1); by (ii) ^1^H NMR (600 MHz, chloroform-*d*): δ 3.69 (s, 3H, CO_2_*CH*_*3*_), 3.26 (dd, *J*=6.5, 4.8 Hz, 1H, H-2), 2.97 (ddd, *J*=11.2, 7.0, 4.2 Hz, 1H, H-6), 2.91–2.83 (m, 1H, *CH*_*2*_CH_2_CN), 2.69 (ddd, *J*=13.3, 7.5, 6.9 Hz, 1H, *CH*_*2*_CH_2_CN), 2.51–2.42 (m, 2H, CH_2_*CH*_*2*_CN), 2.38 (ddd, *J*=11.2, 6.9, 4.3 Hz, 1H, H-6), 1.85–1.74 (m, 2H, H-3), 1.64–1.52 (m, 2H, H-5), 1.55–1.44 (m, 1H, H-4), 1.44–1.35 (m, 1H, H-4) (Supplementary Fig. S2); and (iii) by ^13^C NMR (151 MHz, chloroform-*d*) δ 173.58 (*CO*_*2*_CH_3_), 118.82 (*CN*), 63.68 (C-2), 51.83 (*CH*_*2*_CH_2_CN), 51.59 (CO_2_*CH*_*3*_), 49.55(C-6), 29.24 (C-3), 25.20 [C-5, 21.79 (C-4)], 16.46 (CH_2_*CH*_*2*_CN) (Supplementary Fig. S3).

Next MeNHP was synthesized from *N*-(2-cyanoethyl)-methylpipecolinate. To a stirred solution of the tertiary amine (4.45 g, 22.68 mmol) in DCM p.a. (150 ml) at 0 °C under argon, *m*-CPBA 77% (5.08 g, 22.68 mmol) was added portion wise. Afterwards the mixture was stirred for 1 h at 0 °C and then at room temperature until the reaction was complete (~2 h, controlled by TLC). The solution was diluted with DCM (100 ml) and washed with a saturated aqueous NaHCO_3_ solution (2 × 100 ml) and saline (100 ml). The organic extract was dried over Na_2_SO_4_, filtered, and concentrated. The residue was purified on a flash chromatography column (eluent, hexane EtOAc, 1:1; R_f_, 0.23). The solid product was recrystallized from hexane:EtOAc 10:1, affording a colorless solid (3.05 g, 84%). Its melting point was 80–82 °C. Formation of MeNHP was confirmed by (i) HRMS-ESI: calculated for [C_7_H_13_NO_3_]^+^ (M+H^+^) 160.0968, found 160.0966 and for [C_7_H_13_NO_3_]^+^ (M+Na^+^) 182.0788, found 182.0792 (Supplementary Fig. S4); (ii) ^1^H NMR (400 MHz, DMSO-*d*_6_) δ 8.15 (s, 1H, N-*OH*), 3.61 (s, 3H, CO_2_*CH*_3_), 3.16–3.07 (m, 1H, H-6), 3.00 (d, *J*=11.4 Hz, 1H, H-2), 2.36 (t, *J*=11.7 Hz, 1H, H-6), 1.83–1.70 (m, 1H, H-3), 1.69–1.41 (m, 4H, H-3, H-5, H-4), 1.17 (dt, *J*=12.3, 4.3 Hz, 1H, H-4) and ^1^H NMR (400 MHz, chloroform-*d*) δ 6.46 (s, 1H, N-*OH*), 3.71 (s, 3H, CO_2_*CH*_*3*_), 3.45–3.28 (m, 1H, H-2), 3.14 (d, *J*=11.9 Hz, 1H, H-6), 2.50 (s, 1H, H-6), 1.95 (d, *J*=13.1 Hz, 1H, H-3), 1.83–1.42 (m, 4H, H-3, H-5, H-4), 1.26–1.10 (m, 1H, H-4) (Supplementary Fig. S5); and (iii) ^13^C NMR (101 MHz, DMSO-*d*_6_) δ 172.34 (*CO*_*2*_CH_3_), 70.68 (C-2), 58.05 (C-6), 51.06 (CO_2_*CH*_*3*_), 28.63 (C-3), 24.54 (C-5), 22.17 (C-4) and ^13^C NMR (101 MHz, chloroform-*d*) δ 173.13 (*CO*_*2*_CH_3_), 70.75 (C-2), 57.67 (C-6), 51.93 (CO_2_*CH*_*3*_), 29.42 (C-3), 25.08 (C-5), 22.91 (C-4) (Supplementary Fig. S6).

### Dual-infiltration of authentic NHP and D_9_-NHP standard

Both 1 mM NHP and D_9_-NHP in 10 mM MgCl_2_ were co-infiltrated into three leaves of each individual plant of Col-0, *fmo1-1*, and *ugt76b1-1*. As mock treatment, 10 mM MgCl_2_ was infiltrated alone. Both mock- and NHP-infiltrated plants were either kept without further treatment or were exposed to UV-C radiation for 20 min, as described above. Plants were incubated for 24 h post-treatment. Samples were harvested and stored in –80 °C until extraction with 80% MeOH.

### MeNHP infiltration for metabolite tracking

MeNHP at 1 mM dissolved in 10 mM MgCl_2_ was directly infiltrated into the apical side of the leaf. The infiltrated plants were incubated for 24 h. Leaves were harvested and frozen in liquid nitrogen. The samples were stored at –80 °C.

### MeNHP-induced resistance assay

To investigate the MeNHP-induced resistance, ddH_2_O (mock) or MeNHP at the indicated concentrations diluted in ddH_2_O were infiltrated with a needleless syringe into two full-grown leaves of 3-week-old *fmo1-1* and Col-0 plants. At 24 h post-infiltration, plants were challenged with *H. arabidopsidis* Noco 2 by spraying a conidiaspore solution at a concentration of 50 000 spores ml^–1^. The challenged plants were then grown in a plant chamber at 18 °C with a relative humidity of 80% under a short-day cycle (8 h light/16 h dark). Infection was scored at 7 d post-inoculation as described previously (Ding *et al.*, 2016). In brief, infection was scored by the conidiaspore growth on distal leaves with the following rating category: category 5, >5 conidiaspores observed on more than two distal leaves; category 4, >5 conidiaspores observed on two distal leaves; category 3, <5 conidiaspores observed on two distal leaves; category 2, >5 conidiaspores observed on one distal leaf; category 1, <5 conidiaspores observed on one distal leaf; category 0, no conidiaspores observed on any distal leaves.

### Extraction of plant metabolites

**Fig. 2.**
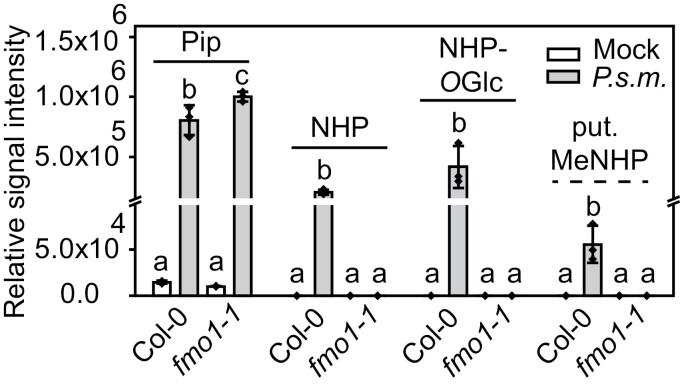
Accumulation of selected FMO1-dependent and independent metabolites in Col-0 and fmo1-1 leaves in response to P.s.m. infiltration. A. thaliana plants grown for 6 weeks under short-day conditions (8 h light/16 h dark) were infiltrated with 10 mM MgCl2 (Mock) or the virulent P. syringae ES4326 strain at OD600=0.05 in 10 mM MgCl2 (P.s.m.). Infiltrated leaves were harvested 24 h post-infiltration, extracted by the MTBE procedure, and analyzed via UHPLC-HRMS. Data were processed using Profinder 8.0 (Agilent Technologies) and OriginPro2020 (Origin). Relative signal intensities of Pip, NHP, NHP-Oglc, and putative methyl-NHP (put. MeNHP) are shown. The y-axis has a break between 1 × 105 and 1.5 × 105. Bars represent mean values with the SD of n=3 replicates. Letters indicate statistical differences, individually calculated for each metabolite (P<0.05, one-way ANOVA followed by post-hoc Tukey test). Each replicate represents an independent pool of nine infiltrated leaves from three plants.

Metabolite extracts were generated from frozen leaf material. Leaves were ground in liquid nitrogen to 100 mg FW [methyl-tert-butyl ether (MTBE) Fig. 2] or 50 mg FW (80% MeOH). The MTBE extraction was performed as described earlier (Mohnike *et al.*, 2021). The 80% MeOH extraction was performed in the following way:. Aliquots (50 mg) of ground leaf material were placed in 2 ml Eppendorf cups and 800 µl of 80% MeOH were added. The samples were vortexed to achieve homogenization. Afterwards, ultrasonication was applied to the samples twice for 15 min. The samples were centrifuged at 18 000 *g* for 15 min. A 700 µl aliquot of debris-free supernatant was transferred into new tubes and evaporated under a stream of nitrogen. Metabolites were resolved in 20% MeOH by vortexing. The solutions were centrifuged at 18 000 *g* for 10 min prior to analysis to remove the remaining debris and 80 µl was transferred into the LC-MS vials.

### UHPLC-HRMS-based metabolite fingerprinting

Metabolite fingerprinting was conducted according to Feussner *et al.* (2022) as described in Mohnike *et al.* (2021). In brief, extracted samples were analyzed with the UHPLC1290 (Agilent Technologies, Santa Clara, CA, USA) coupled to an HRMS instrument 6540 UHD Accurate Mass Q-TOF (Agilent Technologies) with Agilent Dual Jet Stream Technology as the ESI source (Agilent Technologies). An ACQUITY HSS T3 column (2.1 × 100 mm, 1.8 µm particle size, Waters Corporation) was used for chromatographic separation at a flow rate of 500 µl min^–1^ at 40 °C. The solvent system applied was A [water, 0.1% (v/v) formic acid] and B [acetronitrile, 0.1% (v/v) formic acid]. The gradient applied was: 0–3 min, 1–20% B; 3–8 min, 20–97% B; 8–12 min, 10% B; 12–15 min, 1% B. The technical details were described recently (Mohnike *et al.*, 2021). Data were acquired using Mass Hunter Acquisition B.03.01. Data deconvolution was performed using Profinder 10.0 (Agilent Technologies). Data were processed using MarVis-Suite (Kaever *et al.*, 2009, 2012, 2015) (http://marvis.gobics.de) or OriginPro2020 (OriginLab Corporation, Northampton, MA, USA).

## Results

### Identification of MeNHP via metabolite fingerprinting

The major aim of this study was to analyze NHP metabolism upon stress and to identify novel NHP metabolites. Following the hypothesis that molecules of NHP turnover are missing in *fmo1-1* plants, we compared Col-0 wild-type against *fmo1-1* leaves that were infected with *P.s.m.* The leaf extracts were analyzed via UHPLC-HRMS and the obtained dataset was searched for hypothetical NHP metabolites based on previously described modifications of SA and jasmonic acid, such as hydroxylation, dehydrogenation, decarboxylation, and methylation. As proof of concept, the dataset was analyzed for pipecolic acid (Pip), NHP, and NHP-*O*Glc accumulation after *P.s.m.* treatment. As expected, NHP and NHP-*O*Glc were not detected in *fmo1-1* plants (Fig. 2). However, we detected exclusively in Col-0 a relative signal intensity with a mass-to-charge ratio (*m*/*z*) of 160.097 in the positive ionization mode and a retention time of 2.63 min, which corresponds to the mass of methylated-NHP (putative MeNHP). The exact mass of this molecule has been calculated as 159.090 Da (C_7_H_13_NO_3_), showing a mass shift of 14.015 Da compared with NHP. This shift is equivalent to a methyl group derived possibly from methylation of NHP.

### MeNHP is a metabolite of NHP and its identity was unequivocally confirmed by authentic NHP methyl ester standard

**Fig. 3.**
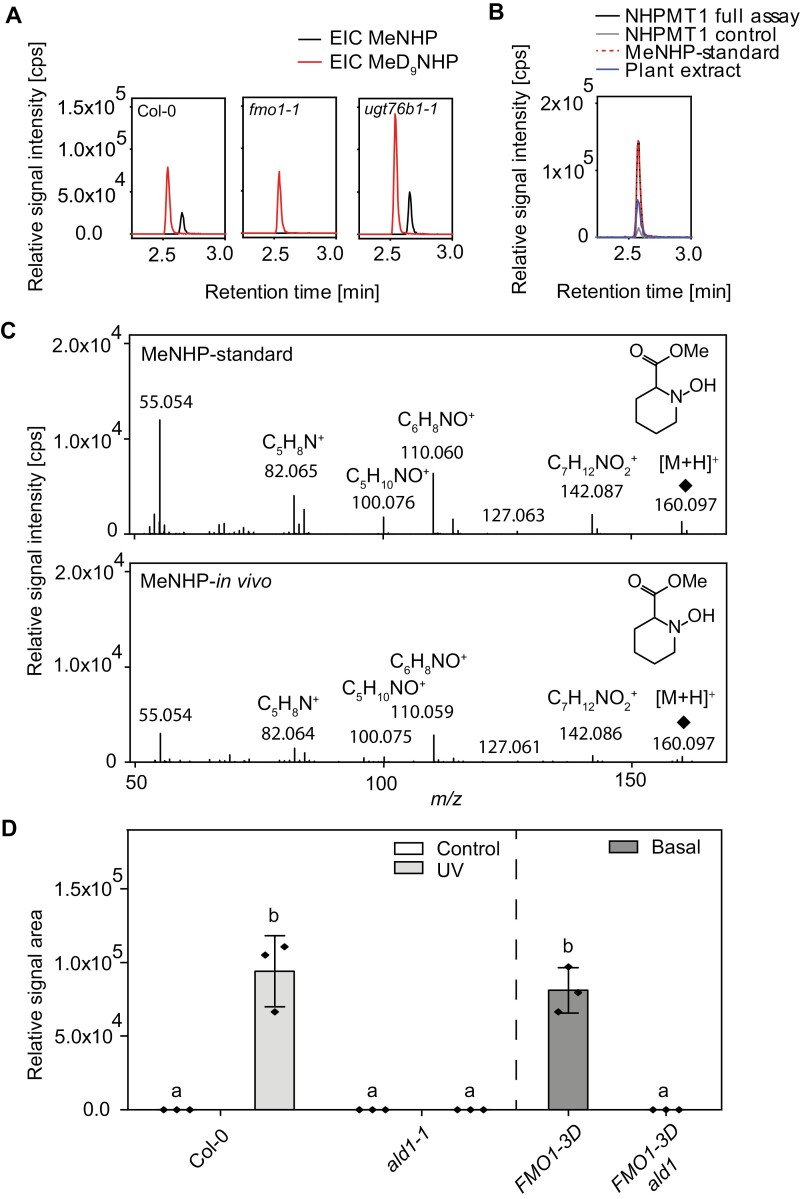
Characterization of N-hydroxy pipecolic acid methyl ester (MeNHP) upon UV-C treatment. (A) Extracted ion chromatograms of MeNHP [M+H]+ 160.097 and D9-MeNHP [M+H]+ 169.153 in Col-0, fmo1-1, and ugt76b1-1 leaves. Col-0, fmo1-1, and ugt76b1-1 leaves were infiltrated with D9-NHP. After 24 h, leaves were harvested, extracted, and analyzed by UHPLC-HRMS. (B) Extracted ion chromatograms at [M+H]+ 160.097 of in vivo metabolite extract, synthesized authentic NHP methyl ester standard, in vitro assay with NHPMT1, and in vitro assay with boiled NHPMT1 as control. (C) Comparison of MS/MS fragments of authentic MeNHP standard and in planta MeNHP. (D) Relative amount of MeNHP in Col-0, ald1, FMO1-3D, and FMO1-3D ald1 plants. Col-0 and ald1 plants were kept untreated or treated with UV-C for 20 min. Plants were incubated for 24 h before harvesting. FMO1-3D and FMO1-3D ald1 plants were left untreated before harvesting. Data represent the mean of three biological replicates for the MeNHP signal (relative signal area) via UHPLC-HRMS analysis. Error bars represent the SD. Letters indicate statistical differences, individually calculated for each experiment (P<0.05, one-way ANOVA followed by post-hoc Tukey test).

In contrast to infection experiments, UV treatment shows much better reproducibility, and UV-C treatment was shown to be an infection-independent tool to elicit pathogen-like defense responses, as shown for SA (Yalpani *et al.*, 1994) as well as for NHP accumulation (Mohnike *et al.*, 2021). In addition, metabolomics data of UV-C-treated leaf material do not interfere with the metabolome of a pathogen. For these reasons, we decided to analyze the MeNHP structure as well as the occurrence of additional NHP-derived metabolites in UV-C-treated leaf material. To confirm MeNHP as a metabolite of NHP, therefore, being dependent on functional *FMO1*, we infiltrated labeled D_9_-NHP into Col-0 and *fmo1-1* leaves and measured formation of D_9_-MeNHP. We detected D_9_-MeNHP in both Col-0 and *fmo1-1* leaves. The labeled compound had a retention time shift towards a polar elution compared with MeNHP. Non-labeled native MeNHP was again only found in Col-0 leaves (Fig. 3A).

Next, we developed a strategy for the chemical synthesis of an NHP methyl ester standard to confirm the identity of MeNHP unequivocally. Additionally, we tried to identify potential methyltransferase candidates with publicly available co-expression data files of the NHP-metabolizing enzyme UGT76B1 (ATTED II, version 11.0). One gene of interest was AT4G22530 [putative NHP-methyl transferase 1 (NHPMT1)] which was annotated as *S*-adenosylmethionine (SAM)-dependent methyl transferase and its expression is NHP responsive (Yildiz *et al.*, 2021). The cDNA of the gene was cloned into a pET28a-expression vector, and the encoded protein was heterologously expressed in *E. coli*, purified to homogeneity, and used for an *in vitro* activity assay with NHP as substrate. The reaction was followed by UHPLC-HRMS (Supplementary Fig. S7). Indeed, the authentic MeNHP standard co-eluted with *in planta* and enzymatically generated MeNHP at a retention time of 2.57 min, and is presented in the extracted ion chromatogram of *m/z* 160.097 (Fig. 3B).

In addition, the fragmentation pattern of the MS/MS spectra of *m/z* 160.097 (MeNHP) exhibits identical fragments to the *in vivo* derived MeNHP and the authentic standard. The main fragments in the positive ionization mode are *m/z* 142.08, *m/z* 127.063, *m/z* 110.060, *m/z* 100.076, and *m/z* 82.065 (mass accuracy of ±2 mDa) (Fig. 3C). The fragment *m/z* 142.08 represents C_7_H_12_NO_2_^+^ after a loss of the *N*-hydroxy group, comparable with NHP fragment *m/z* 127.063 (Supplementary Fig. S8). Moreover, identical fragmentation behavior of MeNHP (C_7_H_13_NO_3_) and NHP (C_6_H_11_NO_3_) is observed by fragment ions *m/z* 110.06, *m/z* 100.07, and *m/z* 82.06. First, *m/z* 110.06 represents C_6_H_8_NO^+^ after loss of two hydroxy groups. Second, *m/z* 100.07 represents the fragment C_5_H_10_NO^+^ obtained by the loss of the carboxylic acid moiety with NHP and the methyl carboxylic acid moiety with MeNHP. Finally, *m/z* 82.06 resembles the fragment of the N-containing hetero ring structure (C_5_H_8_N^+^, dihydropyridine) after loss of the hydroxyl group and carboxylic acid methyl ester group. Together, the structure of the infection-dependent NHP-derived compound MeNHP as an NHP methyl ester was confirmed.

To strengthen the hypothesis that MeNHP is a downstream metabolite of NHP, we investigated the influence of *ald1* loss-of-function mutation on the occurrence of MeNHP *in vivo.* Therefore, we again challenged the plant with UV-C light (Fig. 3D). We observed that MeNHP accumulated in Col-0 plants 24 h post-UV (hpUV) stress. Col-0 control plants and *ald1* control, as well as *ald1* UV-treated plants, did not show any signal for MeNHP at 24 hpUV. Moreover, we tested *FMO1-3D* autoimmune plants and *FMO1-3D ald1* double mutant plants for their basal MeNHP amount (Fig. 3D). We observed a constitutive accumulation of MeMHP in the *FMO1-3D* mutant background, whereas no MeNHP was detected in the *FMO1-3D ald1* background.

In conclusion, these data further support that MeNHP is NHP methyl ester produced *in planta* downstream of NHP. Mutations in the major biosynthetic genes *ald1* and *fmo1* led to absence of MeNHP after stress-induced biosynthesis. Additionally, we showed that NHPMT1 was able to catalyze the formation of MeNHP from NHP and SAM *in vitro* and, therefore, provide additional data that confirm the exact mass and retention time information from *in vivo* MeNHP and the chemically synthesized authentic standard. Whether MeNHP has an influence on plant immunity remains to be investigated. It also remains to be determined whether additional molecules other than MeNHP, NHP-*O*-Glc, and NHP-GE derive from NHP directly and are present in the Pip/NHP molecular network *in vivo*.

### Identification of NHP-derived metabolites via NHP and D_9_-NHP leaf infiltration, UV treatment, and non-targeted metabolomics

To confirm the occurrence of the observed NHP derivative after stress application and to screen for additional NHP derivatives, a non-targeted metabolome experiment was performed as another independent line of evidence. We applied NHP and D_9_-NHP co-infiltration, as well as mock infiltration with 10 mM MgCl_2_ in leaves of wild-type, *fmo1-1*, and *ugt76b1-1* plants, and treated the leaves with UV-C afterwards to stimulate NHP accumulation (Mohnike *et al.*, 2021). The aim of this experiment was to identify all NHP-derived metabolites, by selecting pairwise features with a mass shift of 9.056 Da (exchange of all nine hydrogens of the pyridine moiety by deuterium in NHP) and a small retention time shift of <0.13 min, that are enriched after NHP/D_9_-NHP infiltration, and can ideally be synthesized *in vivo*, without external application. In addition, *ugt76b1-1* mutant plants were included as we hypothesized that other NHP-derived metabolites will accumulate as alternative routes for NHP turnover when NHP-*O*Glc cannot be synthesized.

**Fig. 4.**
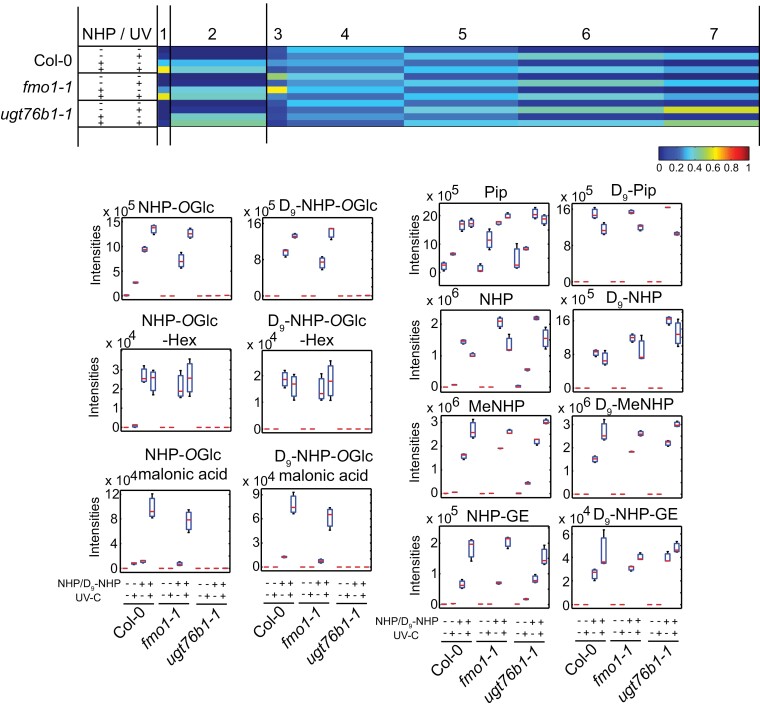
Co-infiltration of NHP/D9-NHP into Col-0, fmo1-1, and ugt76b1-1 with a subsequent UV-C trigger confirms additional in planta synthesis of NHP derivates. Either mock solution (10 mM MgCl2) or a mixture of 1 mM NHP and 1 mM D9-NHP was infiltrated into unstressed leave. Plants were kept untreated as a control or stressed afterwards for 20 min with UV-C light. The plants were incubated for 24 h in short-day conditions before infiltrated leaves were harvested. The extracted leaf material was analyzed with UHPLC-HRMS. Data were analyzed using Profinder 10.0 (Agilent Technologies) and MarVis (Kaever *et al*., 2015). Each sample represents an independent pool of six infiltrated leaves of two plants each. Data are shown in box-plots representing the mean and SD.

The non-targeted metabolite fingerprinting identified 1152 metabolite features with a false discovery rate (FDR) <10^–5^. Overall, these features are arranged by pattern-based clustering via one-dimensional self-organizing maps (1D-SOMs) into seven clusters (Fig. 4; Supplementary Dataset S1). The majority of the metabolite features, represented by cluster numbers 4–7, show a UV treatment-dependent pattern, either depleted (cluster 4) or enriched (clusters 5–7) after UV exposure. In contrast to this, in cluster 1 and 2 metabolite features are represented that mainly show accumulation as a consequence of NHP/D_9_-NHP co-infiltration. Cluster 1 shows metabolites accumulating after NHP infiltration in an *UGT76B1*- as well as a UV-C-dependent manner. Here three pairs of features were detected with a mass shift of 9.056 Da. First, the pair NHP-*O*Glc/D_9_-NHP-*O*Glc was detected in cluster 1, as NHP-*O*Glc is known to be exclusively synthesized by UGT76B1 *in vivo*. This chemotype cannot be restored by external application of NHPs to the *ugt76b1-*1 mutant plants. Furthermore, NHP-*O*Glc also accumulated in those Col-0 samples after UV stress, where NHP/D_9_-NHP infiltration did not take place. As expected, D_9_-NHP-*O*Glc was present in Col-0 and *fmo1-1* plants after NHP infiltration. The second pair of features with a mass shift of 9.056 Da has exact masses of [M+H]^+^/[D_9_M+H]^+^ 470.185/479.241. The exact mass information and its UGT76B1 dependency enabled us to tentatively assign the features as NHP-*O*Glc-Hex/D_9_-NHP-*O*Glc-Hex. Subtracting the exact mass of NHP-*O*Glc ([M+H]^+^ 308.134) from the molecule of [M+H]^+^ 470.185 results in a fragment of 162.051 Da, which corresponds to a hexose moiety. Since the pair of features is exclusively detected in the lines with functional UGT76B1, it strongly suggests that UGT76B1 is responsible for the *O*-glycosylation of NHP required for NHP-*O*Glc-Hex synthesis. In-source fragmentation analysis underlines the compound identity by the detection of NHP-*O*Glc as a fragment ion of [M+H]^+^ 308.134 (Supplementary Fig. S9). In addition, NHP-*O*Glc-Hex is present in mock-infiltrated UV-stressed Col-0 plants, which confirms NHP-*O*Glc-Hex as a native NHP derivative. The third pair of features in cluster 1 fits NHP-*O*Glc/D_9_-NHP-*O*Glc with an additional C_3_H_3_O_3_ moiety, [M+H]^+^/[D_9_M+H]^+^ 394.132/403.188. It is also *UGT76B1* dependent, as the molecular features are not present in the *ugt76b1-1* background. A small amount of NHP-*O*Glc-C_3_H_3_O_3_ in mock-infiltrated, UV-stressed Col-0 plants confirmed that this metabolite is a native NHP derivative. MS/MS fragment analysis of the unknown molecule yielded a fragment of the NHP backbone of [M+H]^+^ 308.134, and suggests an additional malonic acid residue of *m/z* 87.007 as the fragment ion (Supplementary Fig. S10). Together, the MS data led us to assign the third pair of features as NHP-*O*Glc-malonic acid.

Cluster 2 represents metabolites that are enriched in all three genotypes after NHP/D_9_-NHP infiltration, with and without UV-C treatment. Via mass shift search, we detected Pip/D_9_-Pip and NHP/D_9_-NHP, MeNHP/D_9_-MeNHP, and NHP-GE/D_9_-NHP-GE as NHP-derived metabolites. Pip, NHP, and MeNHP accumulated in Col-0 and *ugt76b1-1* after UV stress. In contrast, NHP-GE was detected after UV stress in *ugt76b1-1* and in all plant lines after additional NHP/D_9_-NHP infiltration. Interpretation of the MS/MS fragment pattern confirmed the identity of NHP-GE (Supplementary Fig. S11).

Together the experiment expands the number of novel NHP metabolites to MeNHP, NHP-*O*Glc-Hex, and NHP-*O*Glc-malonic acid. Furthermore, the conversion of D_9_-NHP into D_9_-Pip led us to propose a so far unknown reaction.

### MeNHP application rescues the susceptibility of *fmo1-1* against *H. arabidopsidis* Noco 2 infection

In order to analyze the physiological function of MeNHP, we next analyzed the two mutant alleles *nhpmt1-1* and *nhpmt1-2* as well as one double mutant *nhpmt1-1 ugt76b1-1*. The leaves of all plants were treated with UV-C light again to stimulate NHP accumulation. However, none of the lines showed a reduced MeNHP signal after UV treatment; instead, the signal intensity of NHP and MeNHP was even increased in the analyzed mutants (Supplementary Figs S12, S13).

**Fig. 5.**
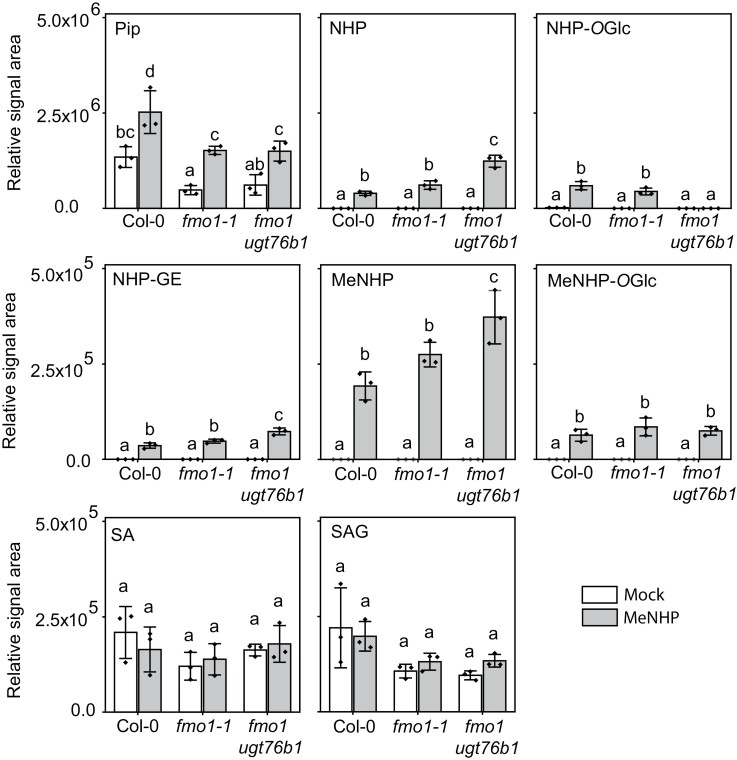
MeNHP infiltration leads to metabolite remodeling including NHP and Pip accumulation in the fmo1-1 mutant background. Col-0, fmo1-1, and fmo1ugt76b1 were grown in short-day conditions for 5 weeks. Plants were infiltrated with 10 mM MgCl2 (Mock) or 1 mM MeNHP in 10 mM MgCl2 (MeNHP). The plants were incubated overnight and harvested 20 h post-infiltration. Samples were extracted with 80% MeOH and measured with UHPLC-HRMS. Data were analyzed using Qualitative Analysis (Agilent Technologies) for the relative signal area of the respective compound shown. Relative signal areas of MeNHP, SA, SAG, Pip, NHP, NHP-OGlc, NHP-GE, and MeNHP-OGlc are shown in mock- and MeNHP-treated Col-0, fmo1-1, and fmo1ugt76b1. Each replicate represents an independent pool of six infiltrated leaves from two plants. Data represent the mean with SD. Statistical analysis was done using Origin Pro 2020. Letters indicate statistical differences (P<0.05, one-way ANOVA followed by post-hoc Tukey test).

Therefore, we decided to analyze next whether the application of MeNHP has an effect on *H. arabidopsidis* Noco 2 infection, and 0.1 mM MeNHP was infiltrated into leaves of Col-0, *fmo1-1*, and *fmo1-1 ugt76b1* plants. Inspired by the observation that SA-binding protein 2 (SABP2) and some of its Arabidopsis orthologs hydrolyze MeSA to SA, we wondered whether MeNHP is hydrolyzed to NHP in Arabidopsis as well (Forouhar *et al.*, 2005; Vlot *et al.*, 2008). In addition, we aimed to determine which other NHP-related metabolites accumulate upon infiltration of MeNHP and whether SA biosynthesis is induced by MeNHP or by MeNHP-derived metabolites. Furthermore, the mutant *fmo1 ugt76b1* was included to identify the origin of MeNHP-*O*Glc detected in our initial experiment described above (Supplementary Fig. S14). After MeNHP infiltration, MeNHP was detected in all three genotypes (Fig. 5), with a higher signal intensity in *fmo1 ugt76b1*. A comparable intensity pattern was observed for NHP, which was significantly enriched after MeNHP treatment in all three backgrounds and accumulated the most in *fmo1 ugt76b1*, which hints at hydrolysis of the infiltrated MeNHP. Interestingly, the relative amount of Pip increased significantly in Col-0, *fmo1-1*, and *fmo1 ugt76b1* plants after MeNHP infiltration in comparison with mock-treated plants. NHP-*O*Glc was not detected in *fmo1 ugt76b1* plants, but significantly accumulates in Col-0 and *fmo1-1*. NHP-GE accumulates in all three backgrounds. Interestingly, we identified a signal of *m/z* 322.149, which may represent MeNHP-*O*Glc. MeNHP-*O*Glc accumulated significantly after MeNHP infiltration independently of *UGT76B1*. To underline the identification, we conducted an enzymatic reaction using the purified SAG-forming enzyme UGT74F1 and were able to reproduce the MeNHP-*O*Glc signal *in vitro* (Supplementary Fig. S14). Furthermore, MeNHP treatment resulted in neither an increase in SA signal nor the accumulation of SAG compared with mock treatment. Similar data were obtained after spraying MeNHP on Col-0 and *fmo1-1* plants (Supplementary Fig. S15). From these results, we conclude that MeNHP can be metabolized in the plant after external application. We were able to detect accumulation of Pip, NHP, NHP-*O*Glc, NHP-GE, and MeNHP-*O*Glc in Col-0, but more importantly in *fmo1-1* knockout plants.

**Fig. 6.**
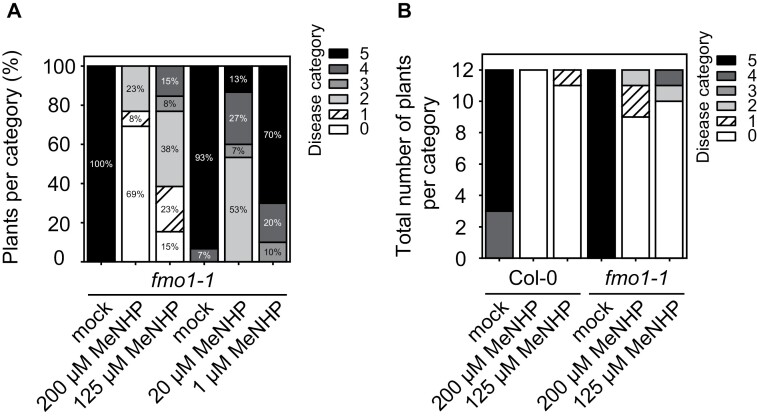
MeNHP infiltration rescues the fmo1-1 infection phenotype against Hyaloperonospora arabidopsidis Noco 2 compared with mock treatment. (A) Four different concentrations of MeNHP (200, 125, 20, and 1 µM) were applied to fmo1-1 mutant plants, and H. arabidopsidis Noco 2 spore growth was assayed compared with individual mock- (water) treated plants. (B) Col-0 and fmo1-1 mutant plants were treated with MeNHP at a concentration of 200 µM or 125 µM. H. arabidopsidis Noco 2 spore growth was assayed compared with individual mock- (water) treated plants. Plants were grouped into disease categories from 5 (heavy spore growth) to 0 (no spore growth). Disease categories were assigned as follows: category 5, >2 leaves harbor >5 spores; 4, two leaves harbor >5 spores; 3, two leaves <5 spores; 2, one leaf >5 spores;1, leaf <5 spores; 0, no spores. n=10–15 plants per treatment.

To test whether MeNHP is able to prime the defense response in Arabidopsis and further to rescue the *fmo1-1* infection phenotype, we challenged MeNHP-treated *fmo1-1* and Col-0 plants with a spore solution of *H. arabidopsidis* Noco 2 and analyzed spore growth on mock- or MeNHP-treated plants (Fig. 6A, B). A concentration gradient of 200, 125, 20, and 1 µM was applied to individual groups of *fmo1-1* mutant plants, and spore growth was analyzed in comparison with mock treatments (Fig. 6A). We assayed two individual mock treatments against either 200 µM and 125 µM or 20 µM and 1 µM MeNHP. With treatment with 200 µM MeNHP, 69% of the pathogen growth was assigned to disease category 0 (no spore growth), 8% to category 1, and 23% to category 2. In the respective mock treatment, growth of the pathogen was grouped into disease category 5 at 100%. The comparison shows reduced pathogen sporulation, and therefore lower disease categories with 200 µM MeNHP treatment compared with mock. The 125 µM MeNHP treatment resulted in a similar trend of reduced pathogen growth. Plants pre-treated with 125 µM MeNHP exhibited no spore growth at 15% and disease categories 1 at 23%, 2 at 38%, 2 at 8%, and 4 at 15%. A comparison between 20 µM MeNHP and mock treatment showed a similar trend to that above. Spore growth on mock-treated plants was assigned to disease categories 4 for 7% and 5 for 93% of plants, whereas spore growth on plants treated with 20 µM MeNHP was grouped into disease categories 2 for 53%, 3 for 7%, 4 for 27%, and 5 for 13%. At the lowest concentration of 1 µM MeNHP, 10% of plants group into category 3, 20% into category 4, and 70% of the challenged plants group into disease category 5. We next applied 200 µM and 125 µM MeNHP to Col-0 and *fmo1-1* plants, respectively, to compare spore growth of *H. arabidopsidis* Noco 2 (Fig. 6B). Mock-treated Col-0 plants group into disease categories 5 and 4. Treatment with 200 µM MeNHP resulted in no spore growth on Col-0. Treatment with 125 µM MeNHP resulted in disease categories 0 and 1. Mock-treated *fmo1-1* mutant plants group into disease category 5. Treatment with 200 µM MeNHP resulted in categories 0, 1, and 2. Application of 125 µM MeNHP resulted in spore growth that was grouped into disease categories 0, 2, and 3.

The experiments confirm that the applied MeNHP is metabolized to NHP in Arabidopsis and that MeNHP treatment is able to rescue the susceptible phenotype of the NHP biosynthesis mutant *fmo1-1* and to induce resistance in Col-0. Furthermore, MeNHP treatment alone does not lead to an increase in signal intensity of SA and SAG; however, the amounts of Pip, NHP, NHP-*O*glc, NHP-GE, and MeNHP-*O*Glc increase significantly.

## Discussion

Intact metabolite networks are key to hormonal balance in plants. In this work, we reveal the NHP metabolome by non-targeted UHPLC-HRMS-based metabolomics upon biotic and abiotic stress. For the initial approach, a non-targeted dataset of Arabidopsis infection with *Pseudomonas* was recorded. The strategy was to identify NHP metabolite features based on exact mass information of the elemental composition. *In silico* modifications were performed, based on well-known metabolizing reactions, such as hydroxylation, methylation, and amino acid conjugation. From the elemental composition of the designed compound, its exact mass was identified and molecular identification was targeted. Via both analysis of *P.s.m.*-infiltrated or UV-C-treated Col-0 and *fmo1-1* mutant plants and dual-infiltration of NHP/D_9_-NHP, three molecules of NHP turnover were identified, namely MeNHP, NHP-*O*Glc-Hex, and NHP-*O*Glc-malonic acid (Figs 2–4). We detected all three metabolites in Col-0, but not in the *fmo1-1* mutant background, after *P.s.m.* infiltration or UV-C treatment. Moreover, we were able to show that MeNHP is metabolized to NHP and that MeNHP treatment is able to rescue the susceptible phenotype of *fmo1-1* mutant plants against *H. arabidopsidis* Noco 2.

### Dual-infiltration as an unbiased method to detect undescribed metabolites of NHP

The dual-infiltration method was developed to overcome the detection limitation of minor metabolites from native plant extracts and to enable unbiased molecular feature identification, independent of a targeted screen. Due to adding both authentic standard and D_9_-labeled authentic standard, the sensitivity of detection of NHP metabolites was increased. In particular, the specificity to pin down a molecule as being of NHP origin was enhanced. By the distinctive mass shift fingerprint and retention time difference, we are able to assign metabolites to NHP origin. Together with the possibility to identify the metabolites in the UV-stressed Col-0 plants, the analysis gives a broad picture of the NHP metabolites. Most importantly, we are able to present molecules absent in the *fmo1-1* background, underlining functional *FMO1* and NHP dependency. NHP metabolites that have already been described are two glycoside forms NHP-*O*Glc and NHP-GE (Chen *et al.*, 2018; Hartmann *et al.*, 2018; Bauer *et al.*, 2021). While the biosynthesis and infection dependency of NHP-*O*Glc have been characterized independently, the unambiguous identification of NHP-GE needs to be confirmed and its route of biosynthesis remains unknown (Bauer *et al.*, 2021; Cai *et al.*, 2021; Holmes *et al.*, 2021; Mohnike *et al.*, 2021). We tested the activity of heterologously expressed and purified UGT73D1 against NHP *in vitro*; however, no NHP-GE synthesizing activity was found (Supplementary Fig. S16). In our analysis, NHP-GE is favorably detected in the *ugt76b1* background but with very low to no abundance in Col-0 plants after *P.s.m.* or UV treatment. As proof of the dual-infiltration concept, we showed the expected molecular pairs of features NHP/D_9_-NHP and NHP-*O*Glc/D_9_-NHP-*O*Glc (Fig. 5). NHP and NHP-*O*Glc are missing in the *fmo1-1* mutant background without external application of NHP/D_9_NHP, but are present when treated with the mixture. Similarly, the MeNHP signal was missing in the *fmo1-1* background, and dual-infiltration restored the MeNHP/D_9_-MeNHP signal. Additionally, we were able to detect *UGT76B1*-dependent NHP metabolites, namely NHP-*O*Glc-Hex and NHP-*O*Glc-Mal. In-source fragment ions underline the identification of NHP-*O*Glc-Hex, as the in-source fragment *m/z* 308.134 represents NHP-*O*Glc (Supplementary Fig. S9). Fragment spectrum analysis suggests malonic acid addition to NHP-*O*Glc represented by a fragment ion at *m/z* 87.008 (Supplementary Fig. S10). Malonic acid moieties at glucose residues are present, for example, in anthocyanins (Bloor and Abrahams, 2002). Interestingly, both molecules are synthesized *in vivo* after UV stress that elicits a similar metabolic response to pathogen infection without the need for additional NHP infiltration. In general, dual-infiltration experiments coupled to non-targeted metabolomics are a valuable approach to track the metabolic course of signal and/or core molecules (Yu *et al.*, 2021).

### Structure elucidation and NHP dependency of MeNHP synthesis

The discovery of the molecular feature at *m/z* 160.097, which was emphasized by the pairwise feature identification in the dual-infiltration experiment, suggested NHP methylation. To confirm MeNHP-detection and to identify its site of methylation, we chemically synthesized NHP methyl ester from pipecolic acid methyl ester. Due to the specificity of methylation at the carboxylic acid group within the MeNHP standard, we were able to exclude hydroxyl methylation. We verified MeNHP as the NHP methyl ester via retention time and MS/MS fragment comparison between the authentic standard and the *in vivo* signal (Fig. 3C). The fragment ions *m/*z 127.063, *m/*z 100.076, and *m/*z 82.065 are identical to NHP fragments, affirming structural similarities and hinting at an NHP-derived molecule (Chen *et al.*, 2018; Hartmann *et al.*, 2018). To strengthen the hypothesis that MeNHP is an NHP-derived metabolite, we analyzed functional dependency on NHP biosynthesis. Via analysis of UV-stressed Col-0 against *ald1* or the basal accumulation of MeNHP in *FMO1-3D* against *FMO1-3D ald1* mutant plants, the dependency of MeNHP on functional NHP biosynthesis was further confirmed (Fig. 3D). In addition, we present an *in vitro* reaction of NHPMT1, which produced MeNHP by using NHP as substrate and SAM as co-substrate. Both *in vivo* and *in vitro*, MeNHP compounds behave as authentic standards with respect to retention time and fragmentation pattern. Despite the *in vitro* activity of NHPMT1 with NHP, *nhpmt1-1*, *nhpmt1-2*, and *nhpmt1-1 ugt76b1-1* mutant plants did not show the absence of MeNHP signal after UV treatment, but the signal intensity of NHP and MeNHP was increased in the analyzed mutants (Supplementary Figs S12, S13). This analysis of *nhpmt1* mutant plants strongly suggests that redundant methyltransferases (MTases) exist for MeNHP synthesis, or that NHPMT1 shows promiscuous *in vitro* MTase activity with NHP but has no influence on *in vivo* synthesis.

### Physiological implications of NHP metabolites

By targeted and non-targeted metabolomics approaches, NHP metabolites were investigated and novel candidate molecules are presented. Additionally, we emphasize the discovery of NHP-GE by Bauer *et al*. and present three novel metabolites which are most probably NHP derived and unambiguously *FMO1* dependent after *P.s.m.* infiltration and UV-C treatment. Independently we present the NHP metabolites in a dual-infiltration study, tracking the metabolic fate via non-targeted UHPLC-HRMS metabolomics. However, their physiological implications remain elusive. We detected accumulation of NHP-GE and MeNHP in the *ugt76b1* background. Taken together, we suggest the carboxy methylation and carboxy glycosylation of NHP as an alternative route of NHP turnover, when *O*-glycosylation is not available.

In analogy to MeSA’s ability to induce systemic resistance in tobacco, we investigated the ability of MeNHP to rescue the *fmo1-1* susceptible phenotype towards an oomycete pathogen (Park *et al.*, 2007; Hartmann *et al.*, 2018). MeSA is proposed to be cleaved by tobacco SABP2. resulting in SA and induced acquired resistance (Forouhar *et al.*, 2005; Park *et al.*, 2007). The molecular structure of SA and NHP opens up the question of whether there is a shared methyl esterase capable of hydrolyzing MeSA and MeNHP in Arabidopsis, similar to their shared mechanism for glucosylation by UGT76B1 (Bauer *et al.*, 2021; Mohnike *et al.*, 2021; Zeier, 2021). In Fig. 5, we show the NHP-related metabolites accumulated upon infiltration of MeNHP. The data suggest a hydrolysis of the externally applied MeNHP to NHP. The external application of MeNHP did not result in significant changes to the SA levels, either in infiltration studies, or after spray application (Fig. 5; Supplementary Fig. S13). Afterwards, we infiltrated MeNHP into *fmo1-1* mutant plants to investigate the potential to enhance disease resistance, aiming in particular to rescue the susceptible phenotype of the *fmo1-1* mutants. We analyzed the spore count of *H. arabidopsidis* Noco 2 on Arabidopsis leaves pre-treated or not with various concentrations of MeNHP (Fig. 6A, B). The data suggest that MeNHP treatment is able to rescue the susceptible phenotype of *fmo1-1* mutant plants, resulting in reduced spore growth. The enhanced resistance after MeNHP treatment at different concentrations might be due to the successful conversion of MeNHP to NHP in the *fmo1-1* background. Furthermore, restoring the NHP pool could be a crucial step to enhance disease resistance in the susceptible *fmo1-1* mutant background, especially since it was shown that NHP rather than Pip is biologically active in the defense response against *Pseudomonas* or oomycete pathogens (Chen *et al.*, 2018; Hartmann *et al.*, 2018). In addition, MeNHP treatment increased Col-0 resistance against *H. arabidopsidis* Noco 2, too. The applied concentrations ranging from 200 µM to 1 µM used for infiltration are within the range (from 1 mM to 1 µM) used in similar studies that infiltrated NHP to induce defense (Chen *et al.*, 2018; Hartmann *et al.*, 2018). Additionally, NHP induces SAR in Arabidopsis in low doses independent of the mode of application (Schnake *et al.*, 2020). Two independent studies underlined the potential of NHP to induce resistance beyond the scope of Arabidopsis (Holmes *et al.*, 2019; Schnake *et al.*, 2020).

**Fig. 7.**
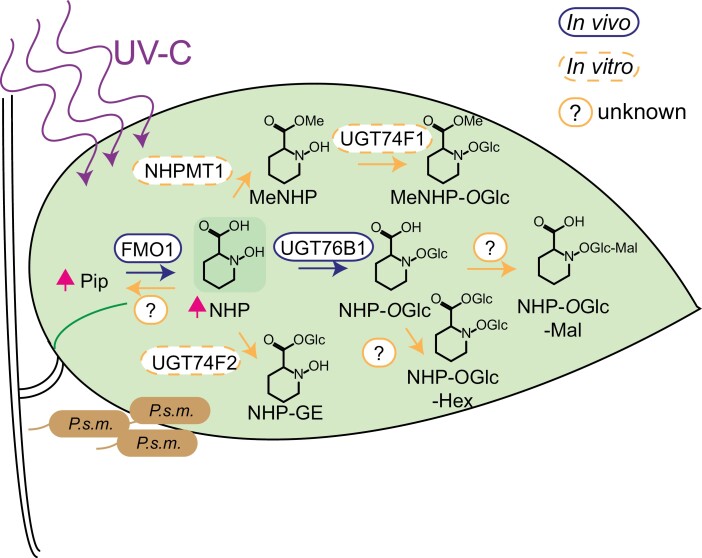
NHP turnover in Arabidopsis induced by UV-C and P.s.m. infiltration. The scheme shows the detected metabolites of NHP turnover. In response to UV-C stress and/or P.s.m. infection, Arabidopsis synthesizes Pip and NHP. To reduce cellular NHP levels, it may be hydrolyzed, glycosylated, and methylated. The respective products were shown to be further metabolized to complex conjugates, NHP-OGlc-Hex and putatively to NHP-OGlc-malonic acid. The potential of MeNHP to induce defense priming was investigated. Further research is required to clarify the role of MeNHP in defense responses, such as in plant–plant communication. Enzymes identified in vivo are circled in purple. Enzymes with identified in vitro activity only are circled in yellow. Reactions for which no enzyme has been described yet are marked with a question mark.

Interestingly we were able to identify another metabolite of MeNHP, when tracking its metabolic fate, namely MeNHP-*O*Hex. This compound is *UGT76B1* independent, suggesting a UGT able to use MeNHP as substrate, conjugating the glucosylation at the *N-*hydroxyl group. *In vitro* reaction using UGT74F1 resulted in reproduction of the MeNHP-*O*Glc signal (Fig. 7; Supplementary Fig. S9). UGT74F1 might be another candidate protein for *in vivo* biosynthesis of the NHP metabolite. Nevertheless, MeNHP-*O*Glc was not shown to be a native product in plant stress response without external application of MeNHP. Hypothetically, UGT71C3 capable of synthesizing MeSA-*O*Glc may be another interesting candidate protein, due to the similarity in structure between MeNHP and MeSA (Chen *et al.*, 2019). We describe that Pip might also be a product of NHP turnover, as not only Pip was accumulating after the dual-infiltration of NHP and D_9_-NHP but also D_9_-Pip. This raises the question of whether there is a reaction to remove the *N*-hydroxylation from NHP via, for example, hydrolases or as an FMO1 reverse reaction. The observation of NHP cleavage may also explain why Pip amounts are still increasing in time-course experiments, when NHP and NHP-*O*Glc are already decreasing in signal intensity (Hartmann and Zeier, 2019).

### Conclusion

Four novel metabolites were identified via UHPLC-HRMS analyses: MeNHP, MeNHP-*O*Glc, NHP-*O*Glc-Hex, and NHP-*O*Glc-Mal after *P.s.m.* infiltration or UV-C treatment (Fig. 7). The potential of MeNHP to induce defense priming was investigated. Further research, however, is required to clarify the role of MeNHP in defense response, for example in plant to plant communication. In addition, metabolites of NHP accumulate in *ugt76b1* mutants, where an important mode of NHP turnover into NHP-*O*Glc is unavailable, suggesting the ability to shuttle NHP into other metabolic pathways to a certain extent.

## Supplementary data

The following supplementary data are available at *JXB* online.

Fig. S1. Mass spectrum of *N*-(2-cyanoethyl)-methylpipecolinate.

Fig. S2. ^1^H-NMR spectrum of *N*-(2-cyanoethyl)-methylpipecolinate.

Fig. S3. ^13^C-NMR spectrum of *N*-(2-cyanoethyl)-methylpipecolinate.

Fig. S4. Mass spectrum of MeNHP.

Fig. S5. ^1^H-NMR spectrum of MeNHP.

Fig. S6. ^13^C-NMR spectrum of MeNHP.

Fig. S7. SDS–PAGE from ion metal affinity chromatography purification of heterologously expressed AT4G22530 (NHPMT1).

Fig. S8. Collision-induced dissociation fragments of MeNHP and NHP

Fig. S9. In-source fragments of the NHP-*O*Glc-Hex and D_9_-NHP-*O*Glc-Hex pair.

Fig. S10. Collision-induced dissociation fragments of the NHP-*O*Glc-malonic acid and D_9_-NHP-*O*glc-malonic acid pair.

Fig. S11. Collision- induced dissociation fragments of NHP-GE.

Fig. S12. Metabolite analysis of UV-treated Col-0 and *nhpmt1* mutant plants.

Fig. S13. MeNHP analysis of UV-stressed Col-0 versus *ugt76b1-1 nhpmt1-1*.

Fig. S14. Infiltration of MeNHP leads to MeNHP-*O*Glc formation, which is underlined *in vitro*.

Fig. S15. Metabolite analysis after spray application of MeNHP.

Fig. S16. UGT73D1 is not active with NHP *in vitro*.

Dataset S1. UHPLC-HRMS-data of NHP/D_9_NHP dual-infiltration.

erac422_suppl_Supplementary_Dataset_S1

erac422_suppl_Supplementary_Figures

## Data Availability

All data generated or analyzed during this study are included in this published article and its supplementary data online.

## Abbreviations

ALD1: AGD2-LIKE DEFENSE RESPONSE PROTEIN 1
*m*-CPBA: *m*-chloroperoxybenzoic acid
CRISPR: clustered regularly interspaced short palindromic repeats
DCM: dichloromethane
1D-SOMs: one-dimensional-self organizing map
EPS1: ENHANCED PSEUDOMONAS SUSCEPTIBILITY 1
ESI: electrospray ionization
Et_3_N: triethylamine
Et_2_O: diethyl ether
EtOAc: ethyl acetate
FMO1: FLAVINE-DEPENDENT MONOOXYGENASE 1
hpUV: hours post-UV light treatment
HR: high resolution
MeNHP: *N*-hydroxy pipecolic acid methyl ester
MeNHP-*O*Glc: MeNHP glycoside
MeOH: methanol
MeSA: salicylic acid methyl ester
MTBE: methyl-*tert*-butyl ether
NHP: *N*-hydroxy pipecolic acid
NHP-*O*Glc: NHP glucoside
NHP-*O*Glc-Hex: NHP glucoside hexose
NHP-GE: NHP glycosyl ester
NHPMT: NHP methyl transferase
PBS3: AvrPphB SUSCEPTIBLE 3
Pip: pipecolic acid
P.s.m.: *Pseudomonas syringae* ES4326
RT: retention time
SA: salicylic acid
SABP2: SA-binding protein 2
SAG: salicylic acid glucoside
SARD4: SYSTEMIC ACQUIRED RESISTANCE DEFICIENT 4
UGT: UDP-dependent glycosyl transferase
UNK: unknown metabolite
UHPLC-HRMS: ultra-high-performance liquid chromatography high-resolution mass spectrometry

## References

[CIT0001] Bauer S , Mekonnen DW, Hartmann M, Yildiz I, Janowski R, Lange B, Geist B, Zeier J, Schäffner AR. 2021. UGT76B1, a promiscuous hub of small molecule-based immune signaling, glucosylates N-hydroxypipecolic acid and balances plant immunity. The Plant Cell 33, 714–734. 33955482 10.1093/plcell/koaa044 PMC8136890

[CIT0002] Bloor SJ , Abrahams S. 2002. The structure of the major anthocyanin in *Arabidopsis thaliana*. Phytochemistry 59, 343–346. 11830144 10.1016/s0031-9422(01)00460-5

[CIT0003] Cai J , Jozwiak A, Holoidovsky L, Meijler MM, Meir S, Rogachev I, Aharoni A. 2021. Glycosylation of *N*-hydroxy-pipecolic aid equilibrates between systemic acquired resistance response and plant growth. Molecular Plant 14, 440–455. 33387676 10.1016/j.molp.2020.12.018

[CIT0004] Chen F , D’Auria JC, Tholl D, Ross JR, Gershenzon J, Noel JP, Pichersky E. 2003. An *Arabidopsis thaliana* gene for methylsalicylate biosynthesis, identified by a biochemical genomics approach, has a role in defense. The Plant Journal 36, 577–588. 14617060 10.1046/j.1365-313x.2003.01902.x

[CIT0005] Chen L , Wang W-s, Wang T, Meng X-f, Chen T-t, Huang X-x, Li Y-J, Hou B-k. 2019. Methyl salicylate glucosylation regulates plant defense signalling and systemic acquired resistance. Plant Physiology 180, 2167–2181. 30962291 10.1104/pp.19.00091 PMC6670094

[CIT0006] Chen Y-C , Holmes EC, Rajniak J, Kim J-G, Tang S, Fischer CR, Mudgett MB, Sattely ES. 2018. *N*-hydroxy-pipecolic acid is a mobile metabolite that induces systemic disease resistance in *Arabidopsis*. Proceedings of the National Academy of Sciences, USA 115, E4920–E4929. 10.1073/pnas.1805291115 PMC6003486 29735713

[CIT0007] Dean JV , Delaney SP. 2008. Metabolism of salicylic acid in wild-type, *ugt74f1* and *ugt74f2* glucosyltransferase mutants of *Arabidopsis thaliana*. Physiologica Plantarum 132, 417–425. 10.1111/j.1399-3054.2007.01041.x 18248508

[CIT0008] Ding P , Rekhter D, Ding Y, et al. 2016. Characterization of a pipecolic acid biosynthesis pathway required for systemic acquired resistance. The Plant Cell 28, 2603–2615. 27758894 10.1105/tpc.16.00486 PMC5134984

[CIT0009] Feussner K , Abreu IN, Klein M, Feussner I. 2022. Metabolite fingerprinting: a powerful metabolomics approach for marker identification and functional gene annotation. Methods in Enzymology, doi: 10.1016/bs.mie.2022.08.015. 36710017

[CIT0010] Forouhar F , Yang Y, Kumar D, et al. 2005. Structural and biochemical studies identify tobacco SABP2 as a methyl salicylate esterase and implicate it in plant innate immunity. Proceedings of the National Academy of Sciences, USA 102, 1773–1778. 10.1073/pnas.0409227102 PMC547883 15668381

[CIT0011] Fu ZQ , Dong X. 2013. Systemic acquired resistance: turning local infection into global defense. Annual Review of Plant Biology 64, 839–863. 10.1146/annurev-arplant-042811-105606 23373699

[CIT0012] Gaffney T , Friedrich L, Vernooij B, Negrotto D, Nye G, Uknes S, Ward E, Kessmann H, Ryals J. 1993. Requirement of salicylic acid for the induction of systemic acquired resistance. Science 261, 754–756. 17757215 10.1126/science.261.5122.754

[CIT0013] George Thompson AM , Iancu CV, Neet KE, Dean JV, Choe JY. 2017. Differences in salicylic acid glucose conjugations by UGT74F1 and UGT74F2 from *Arabidopsis thaliana*. Scientific Reports 7, 46629. 28425481 10.1038/srep46629 PMC5397973

[CIT0014] Haroth S , Feussner K, Kelly AA, Zienkiewicz K, Shaikhqasem A, Herrfurth C, Feussner I. 2019. The glycosyltransferase UGT76E1 significantly contributes to 12-*O*-glucopyranosyl-jasmonic acid formation in wounded *Arabidopsis thaliana* leaves. The Journal of Biological Chemistry 294, 9858–9872. 31072871 10.1074/jbc.RA119.007600 PMC6597828

[CIT0015] Hartmann M , Zeier J. 2018. l-Lysine metabolism to *N*-hydroxypipecolic acid: an integral immune-activating pathway in plants. The Plant Journal 96, 5–21. 30035374 10.1111/tpj.14037

[CIT0016] Hartmann M , Zeier J. 2019. *N*-Hydroxypipecolic acid and salicylic acid: a metabolic duo for systemic acquired resistance. Current Opinion in Plant Biology 50, 44–57. 30927665 10.1016/j.pbi.2019.02.006

[CIT0017] Hartmann M , Zeier T, Bernsdorff F, et al. 2018. Flavin monooxygenase-generated *N*-hydroxypipecolic acid is a critical element of plant systemic immunity. Cell 173, 456–469. 29576453 10.1016/j.cell.2018.02.049

[CIT0018] Holmes EC , Chen Y-C, Mudgett MB, Sattely ES. 2021. Arabidopsis UGT76B1 glycosylates *N*-hydroxy-pipecolic acid and inactivates systemic acquired resistance in tomato. The Plant Cell 33, 750–765. 33955491 10.1093/plcell/koaa052 PMC8136894

[CIT0019] Holmes EC , Chen Y-C, Sattely ES, Mudgett MB. 2019. An engineered pathway for *N*-hydroxy-pipecolic acid synthesis enhances systemic acquired resistance in tomato. Science Signaling 12, eaay3066. 31641079 10.1126/scisignal.aay3066 PMC7954083

[CIT0020] Kaever A , Landesfeind M, Feussner K, Mosblech A, Heilmann I, Morgenstern B, Feussner I, Meinicke P. 2015. MarVis-Pathway: integrative and exploratory pathway analysis of non-targeted metabolomics data. Metabolomics 11, 764–777. 25972773 10.1007/s11306-014-0734-y PMC4419191

[CIT0021] Kaever A , Landesfeind M, Possienke M, Feussner K, Feussner I, Meinicke P. 2012. MarVis-Filter: ranking, filtering, adduct and isotope correction of mass spectrometry data. Journal of Biomedicine and Biotechnology 2012, 263910. 22550397 10.1155/2012/263910 PMC3328170

[CIT0022] Kaever A , Lingner T, Feussner K, Göbel C, Feussner I, Meinicke P. 2009. MarVis: a tool for clustering and visualization of metabolic biomarkers. BMC Bioinformatics 10, 92. 19302701 10.1186/1471-2105-10-92 PMC2666665

[CIT0023] Karasov TL , Chae E, Herman JJ, Bergelson J. 2017. Mechanisms to mitigate the trade-off between growth and defense. The Plant Cell 29, 666–680. 28320784 10.1105/tpc.16.00931 PMC5435432

[CIT0024] Maksym RP , Ghirardo A, Zhang W, von Saint Paul V, Lange B, Geist B, Hajirezaei M-R, Schnitzler J-P, Schäffner AR. 2018. The defense-related isoleucic acid differentially accumulates in *Arabidopsis* among branched-chain amino acid-related 2-hydroxy carboxylic acids. Frontiers in Plant Science 9, 766. 29937770 10.3389/fpls.2018.00766 PMC6002512

[CIT0025] Mohnike L , Rekhter D, Huang W, Feussner K, Tian H, Herrfurth C, Zhang Y, Feussner I. 2021. The glycosyltransferase UGT76B1 modulates *N*-hydroxy-pipecolic acid homeostasis and plant immunity. The Plant Cell 33, 735–749. 33955489 10.1093/plcell/koaa045 PMC8136917

[CIT0026] Navarova H , Bernsdorff F, Döring A-C, Zeier J. 2012. Pipecolic acid, an endogenous mediator of defense amplification and priming, is a critical regulator of inducible plant immunity. The Plant Cell 24, 5123–5141. 23221596 10.1105/tpc.112.103564 PMC3556979

[CIT0027] Noutoshi Y , Okazaki M, Kida T, et al. 2012. Novel plant immune-priming compounds identified via high-throughput chemical screening target salicylic acid glucosyltransferases in *Arabidopsis*. The Plant Cell 24, 3795–3804. 22960909 10.1105/tpc.112.098343 PMC3480303

[CIT0028] Park S-W , Kaimoyo E, Kumar D, Mosher S, Klessig DF. 2007. Methyl salicylate is a critical mobile signal for plant systemic acquired resistance. Science 318, 113–116. 17916738 10.1126/science.1147113

[CIT0029] Rekhter D , Lüdke D, Ding Y, Feussner K, Zienkiewicz K, Lipka V, Wiermer M, Zhang Y, Feussner I. 2019. Isochorismate-derived biosynthesis of the plant stress hormone salicylic acid. Science 365, 498–502. 31371615 10.1126/science.aaw1720

[CIT0030] Schnake A , Hartmann M, Schreiber S, Malik J, Brahmann L, Yildiz I, von Dahlen J, Rose LE, Schaffrath U, Zeier J. 2020. Inducible biosynthesis and immune function of the systemic acquired resistance inducer *N*-hydroxypipecolic acid in monocotyledonous and dicotyledonous plants. Journal of Experimental Botany 71, 6444–6459. 32725118 10.1093/jxb/eraa317 PMC7586749

[CIT0031] Sousa CAD , Sampaio-Dias IE, García-Mera X, Lima CFRAC, Rodríguez-Borges JE. 2016. On the scope of oxidation of tertiary amines: Meisenheimer rearrangements versus Cope elimination in 2-(cyanoethyl)-2-azanorbornanes. Organic Chemistry Frontiers 3, 1624–1634.

[CIT0032] Torrens-Spence MP , Bobokalonova A, Carballo V, Glinkerman CM, Pluskal T, Shen A, Weng J-K. 2019. PBS3 and EPS1 complete salicylic acid biosynthesis from isochorismate in *Arabidopsis*. Molecular Plant 12, 1577–1586. 31760159 10.1016/j.molp.2019.11.005

[CIT0033] Vlot AC , Klessig DF, Park S-W. 2008. Systemic acquired resistance: the elusive signal(s). Current Opinion in Plant Biology 11, 436–442. 18614393 10.1016/j.pbi.2008.05.003

[CIT0034] von Saint Paul V , Zhang W, Kanawati B, Geist B, Faus-Kessler T, Schmitt-Kopplin P, Schäffner AR. 2011. The *Arabidopsis* glucosyltransferase UGT76B1 conjugates isoleucic acid and modulates plant defense and senescence. The Plant Cell 23, 4124–4145. 22080599 10.1105/tpc.111.088443 PMC3246326

[CIT0035] Wildermuth MC , Dewdney J, Wu G, Ausubel FM. 2001. Isochorismate synthase is required to synthesize salicylic acid for plant defence. Nature 414, 562–565. 11734859 10.1038/35107108

[CIT0036] Yalpani N , Enyedi AJ, León J, Raskin I. 1994. Ultraviolet light and ozone stimulate accumulation of salicylic acid, pathogenesis-related proteins and virus resistance in tobacco. Planta 193, 372–376.

[CIT0037] Yildiz I , Mantz M, Hartmann M, Zeier T, Kessel J, Thurow C, Gatz C, Petzsch P, Köhrer K, Zeier J. 2021. Mobile SAR signal *N*-hydroxypipecolic acid induces NPR1-dependent transcriptional reprogramming and immune priming. Plant Physiology 186, 1679–1705. 33871649 10.1093/plphys/kiab166 PMC8260123

[CIT0038] Yu Y , Zhang YK, Manohar M, et al. 2021. Nematode signaling molecules are extensively metabolized by animals, plants, and microorganisms. ACS Chemical Biology 16, 1050–1058. 34019369 10.1021/acschembio.1c00217 PMC8590397

[CIT0039] Zeier J. 2021. Metabolic regulation of systemic acquired resistance. Current Opinion in Plant Biology 62, 102050. 34058598 10.1016/j.pbi.2021.102050

[CIT0040] Zhang K , Halitschke R, Yin C, Liu C-J, Gan S-S. 2013. Salicylic acid 3-hydroxylase regulates Arabidopsis leaf longevity by mediating salicylic acid catabolism. Proceedings of the National Academy of Sciences, USA 110, 14807–14812. 10.1073/pnas.1302702110 PMC3767541 23959884

[CIT0041] Zhang Y , Zhao L, Zhao J, Li Y, Wang J, Guo R, Gan S, Liu C-J, Zhang K. 2017. *S5H/DMR6* encodes a salicylic acid 5-hydroxylase that fine-tunes salicylic acid homeostasis. Plant Physiology 175, 1082–1093. 28899963 10.1104/pp.17.00695 PMC5664462

