# Supplementary material for: N-Hydroxy pipecolic acid methyl ester is involved in Arabidopsis immunity
Source: J Exp Bot. 2022 Oct 19;74(1):458–71. doi: 10.1093/jxb/erac422 (PMC9786843; doi:10.1093/jxb/erac422)
Supplement: erac422_suppl_Supplementary_Figures [file erac422_suppl_supplementary_figures.pdf]

Supplemental Figures

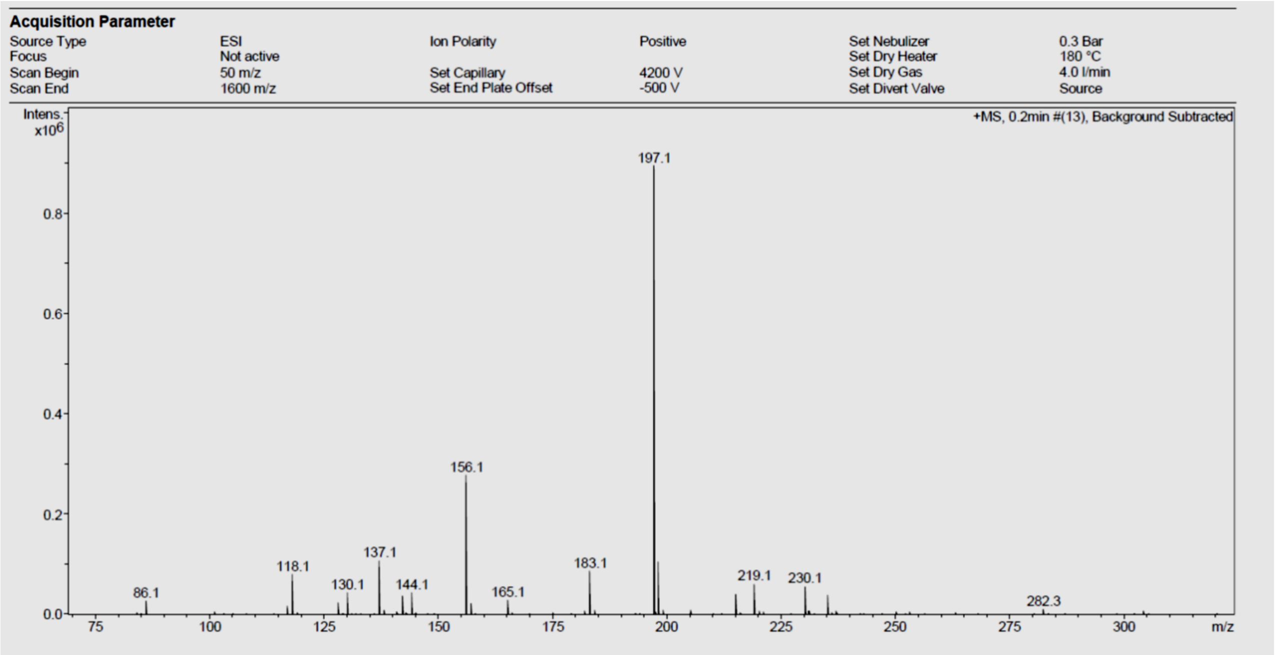

Fig. S1. Mass spectrum of *N*-(2-cyanoethyl)-methylpipecolinate.

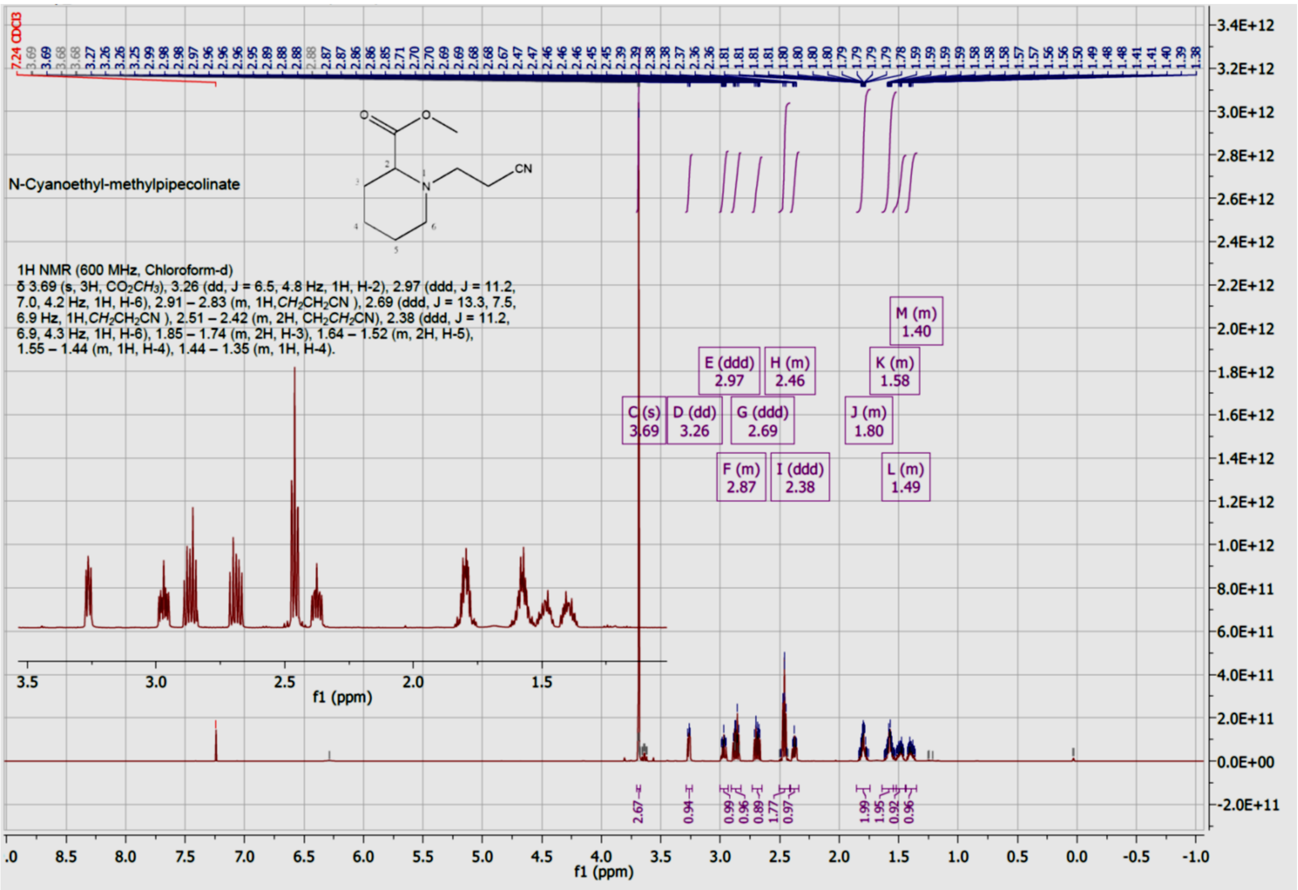

Fig. S2. ^{1}H-NMR spectrum of *N*-(2-cyanoethyl)-methylpipecolinate.

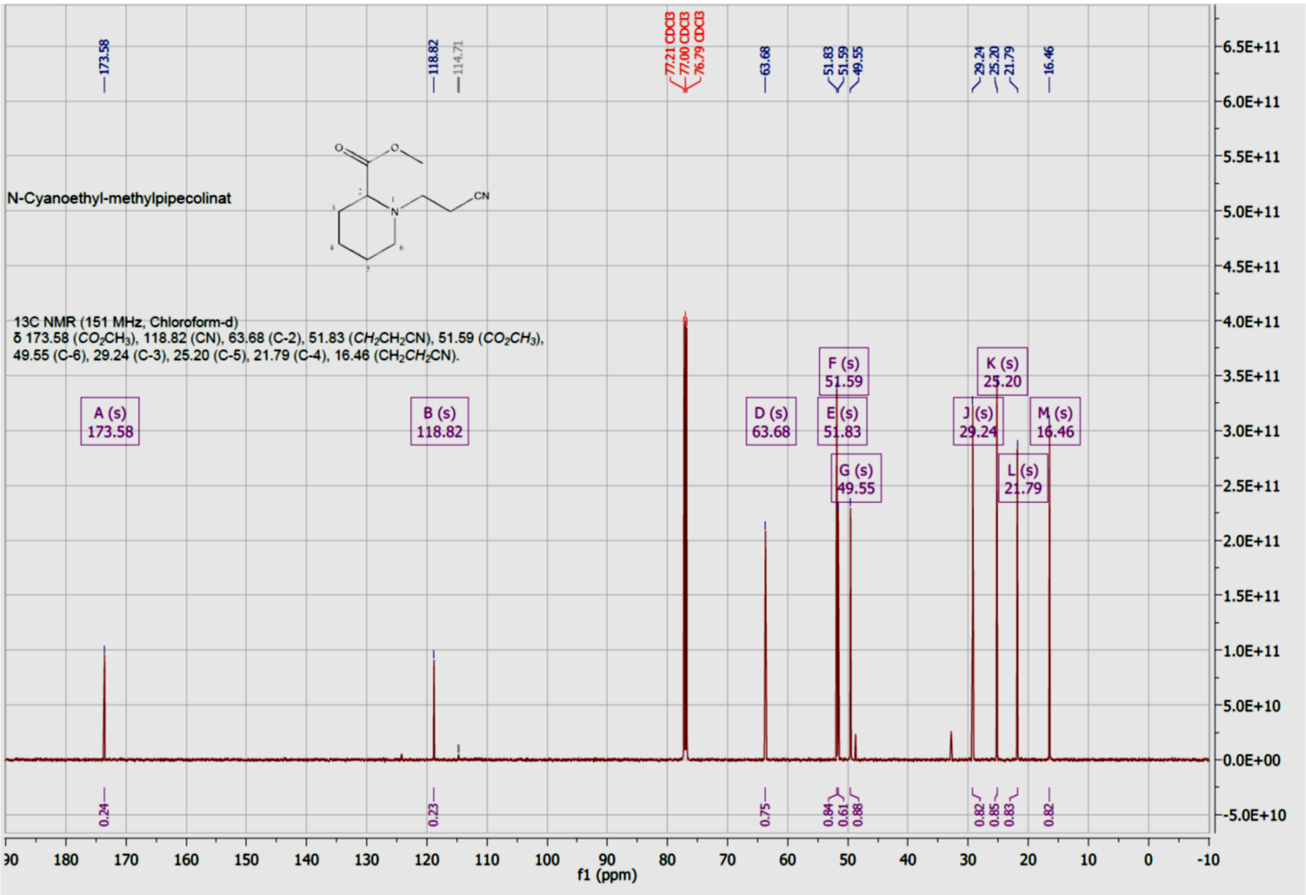

Fig. S3. ^{13}C-NMR spectrum of *N*-(2-cyanoethyl)-methylpipecolinate.

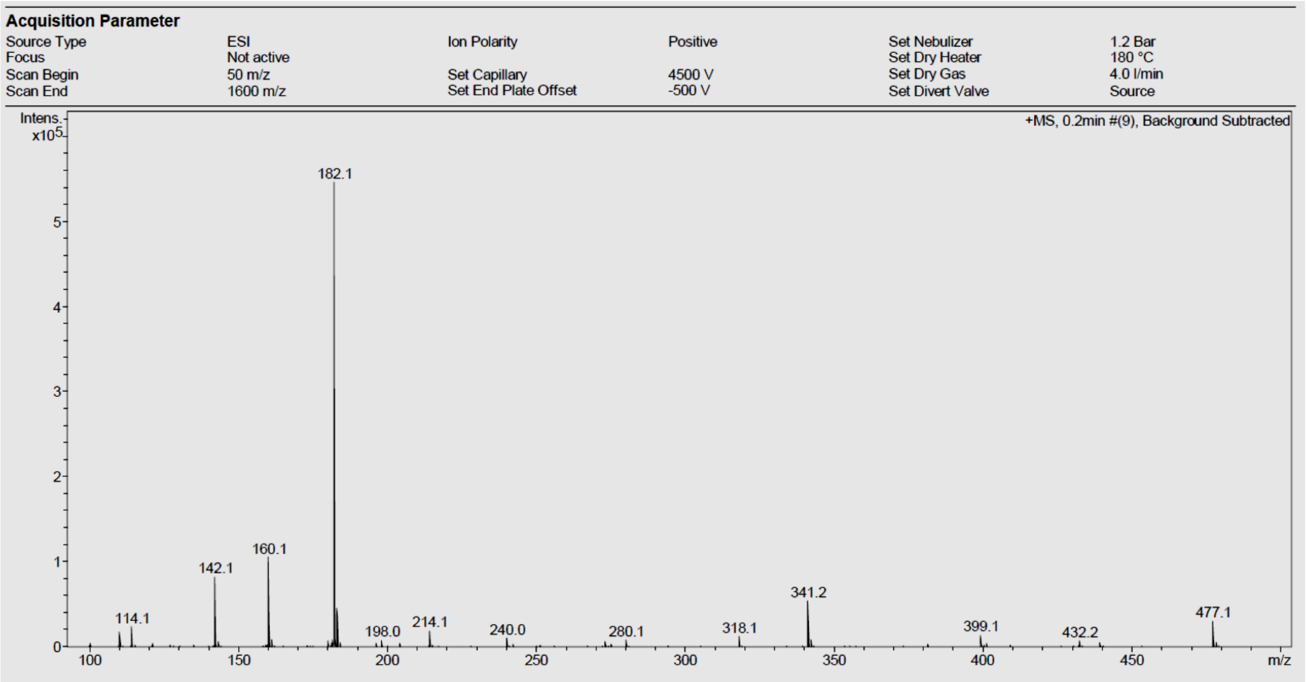

Fig. S4. Mass spectrum of MeNHP.

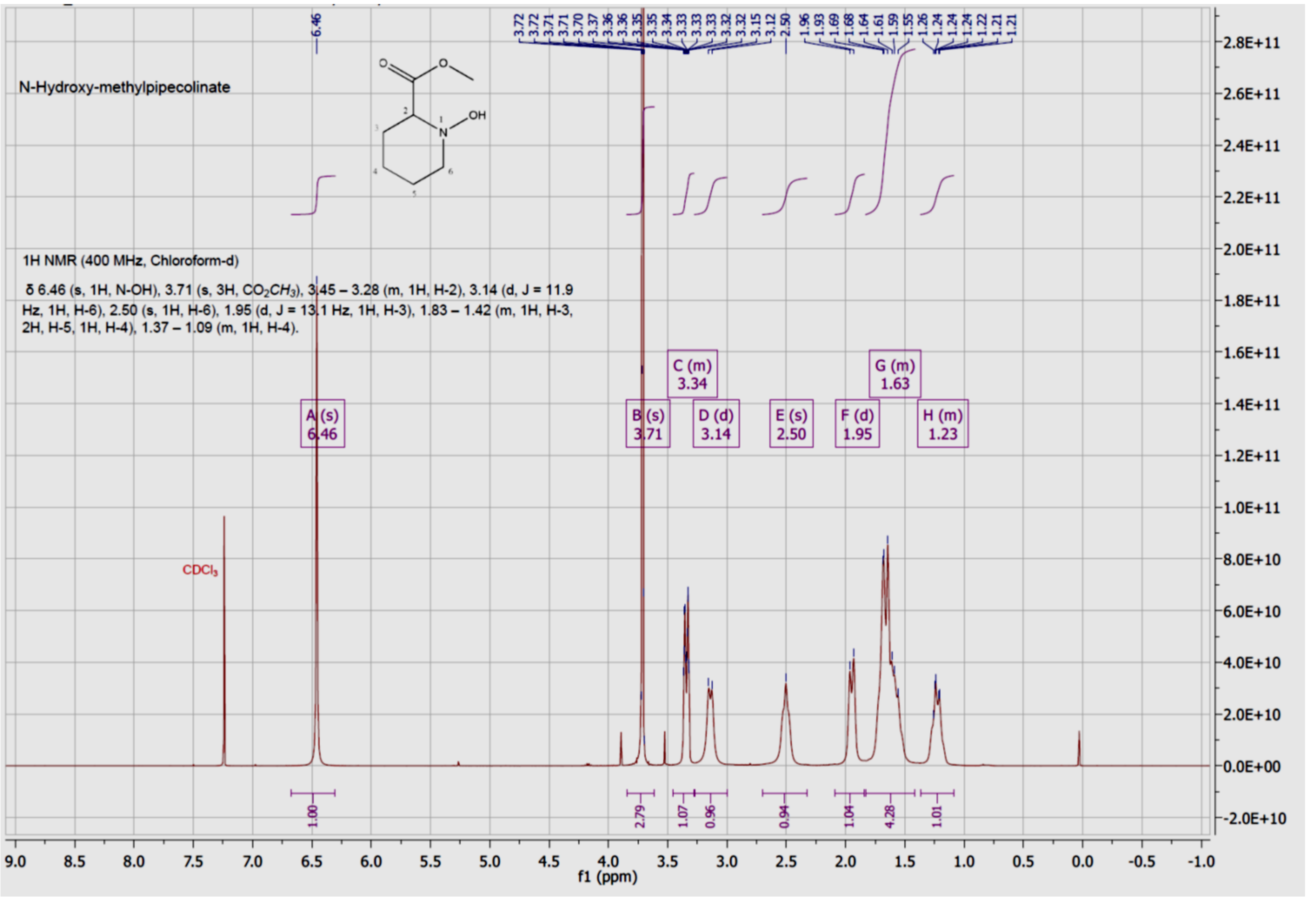

Fig. S5. ^{1}H-NMR spectrum of MeNHP.

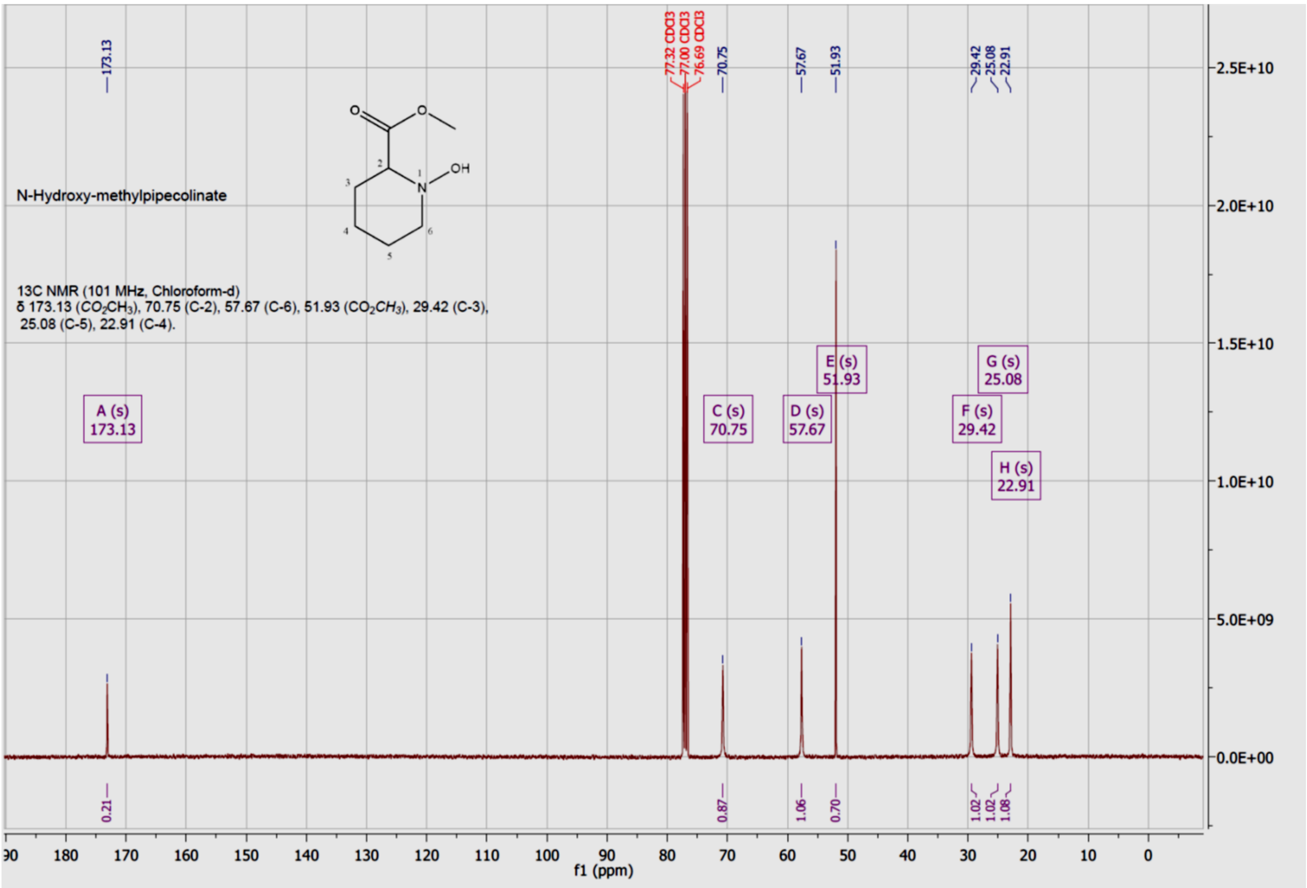

Fig. S6. ^{13}C-NMR spectrum of MeNHP.

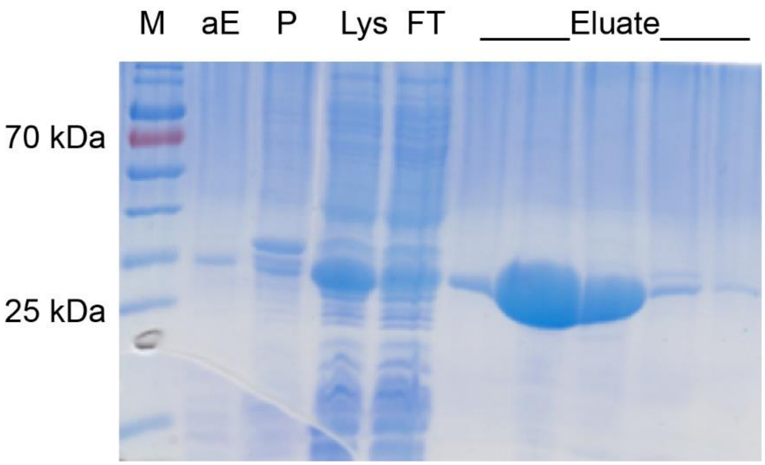

Fig. S7. SDS-PAGE from ion metal affinity chromatography purification of heterologous expressed AT4G22530 (NHPMT1).

From the left fractions represent marker (M), after expression (aE), pellet (P), lysate (Lys), flow through (FT) and combined eluate fractions according to the chromatographic signal of the purification at 280 nm absorption.

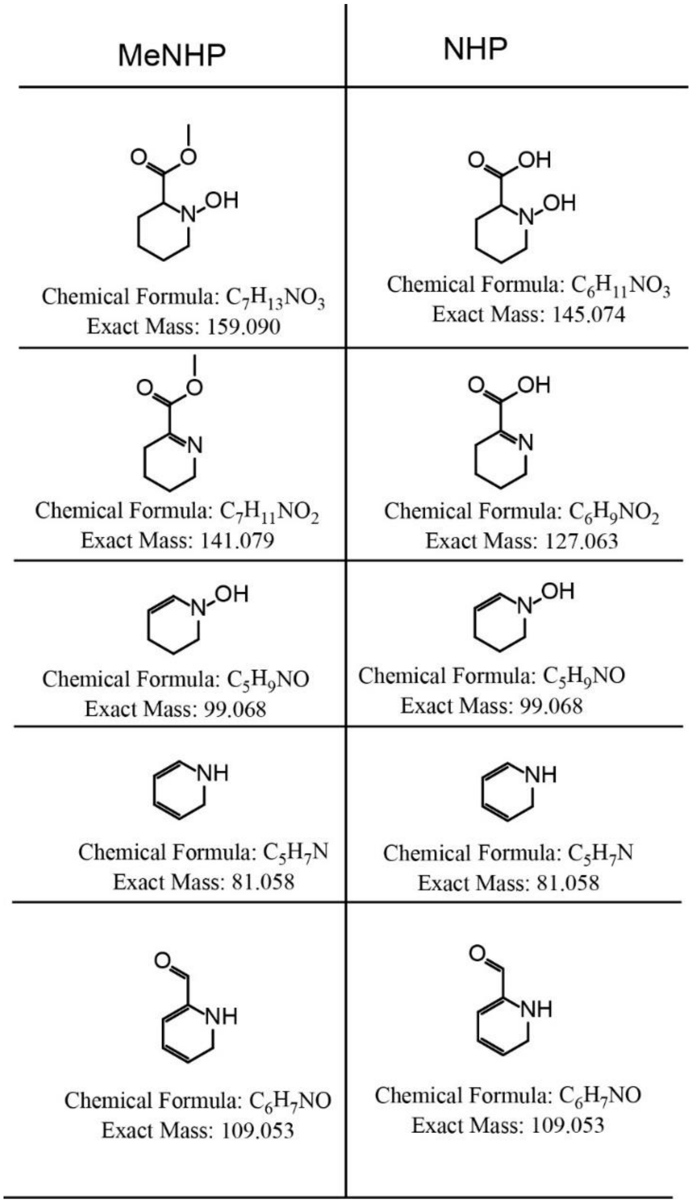

Fig. S8. Collision induced dissociation fragments of MeNHP and NHP.

Mass spectrometric signals according to MeNHP and NHP from *in vivo* metabolite extracts were subject to collision induced dissociation at 10 eV. Fragments were analyzed with high-resolution mass spectrometry. A unique fragment of MeNHP was $C_7H_{11}NO_2$ with a deduced neutral mass of 141.079 Da. This fragment is equivalent to the unique NHP fragment of $C_6H_9NO_2$ with a deduced neutral mass of 127.063 Da. The fragments represent loss of the *N*-hydroxy moiety. Identical fragments of both molecules show the formula C_6H_7NO. It arises by loss of two water and has been interpreted as loss of the *N*-hydroxy groups and loss of water derived from the carboxy function of NHP. Next, the fragment of C_5H_9NO occurs by loss of the carboxylic acid group. The fragment of C_5H_7N derived from loss of carboxylic acid and *N*-hydroxy group).

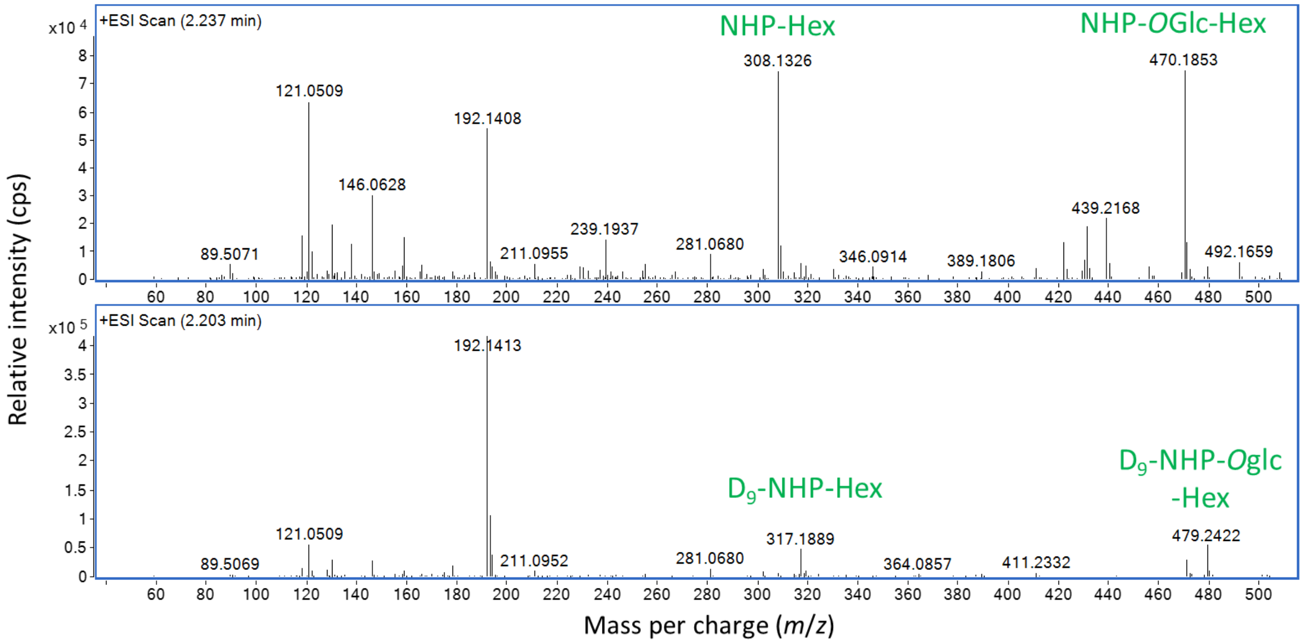

Fig. S9. Insource fragments of NHP-*O*Glc-Hex and D_9-NHP-*O*Glc-Hex feature pair.

Mass spectrometric signals according to *m/z* 470.185/479.242 from *in vivo* metabolite extracts were analyzed on in-source fragmentation. Fragments were analyzed with high-resolution mass spectrometry. NHP-Hex and D_9-NHP-Hex fragments were identified as in-source fragments underlining the identification.

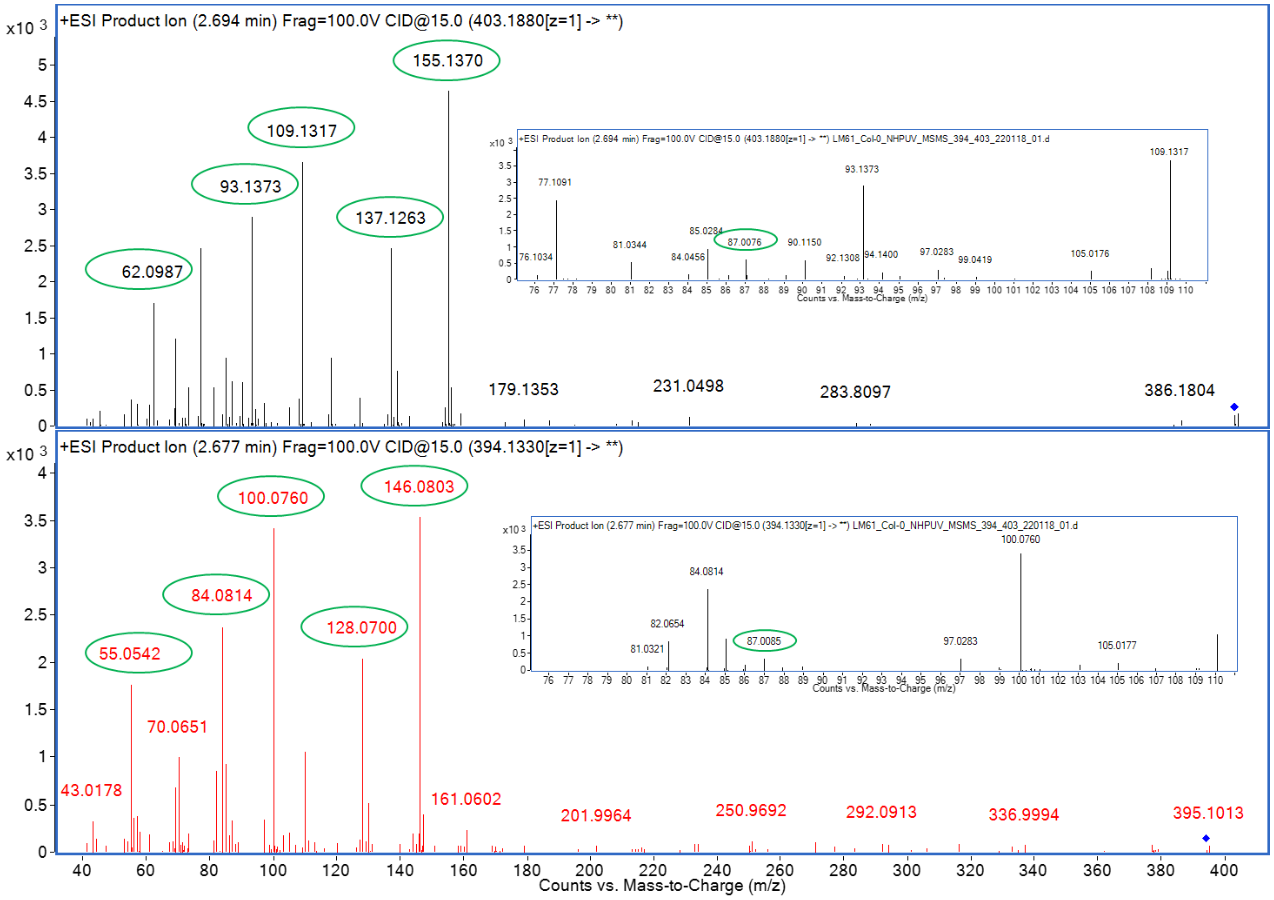

Fig. S10. Collision induced dissociation fragments of NHP-*O*Glc-malonic acid and D_9-NHP-*O*Glc malonic acid feature pair.

Mass spectrometric signals according to *m/z* 394.133/403.2188 from *in vivo* metabolite extracts were subject to collision induced dissociation at 15 eV. Fragments were analyzed with high-resolution mass spectrometry. NHP and D_9-NHP fragments were identified. Additionally, a fragment of *m/z* 87.007 was identified in both spectra, which could represent a malonic acid fragment (inserted spectra).

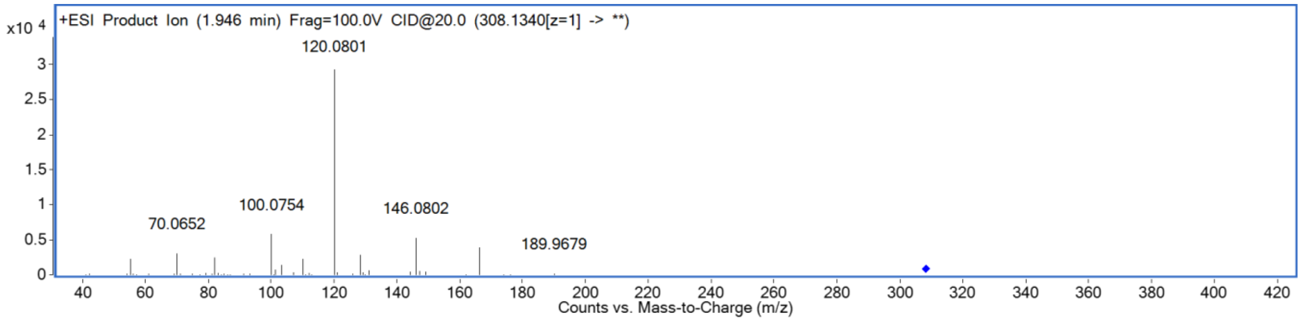

Fig. S11. Collision induced dissociation fragments of NHP-GE.

Mass spectrometric signals according to *m/z* 308.134 from *in vivo* metabolite extract of *ugt76b1* plants that were soil drenched with 10 mM NHP. The NHP-GE signal was subject to collision induced dissociation at 20 eV. Fragments were analyzed with high-resolution mass spectrometry. NHP fragments were identified.

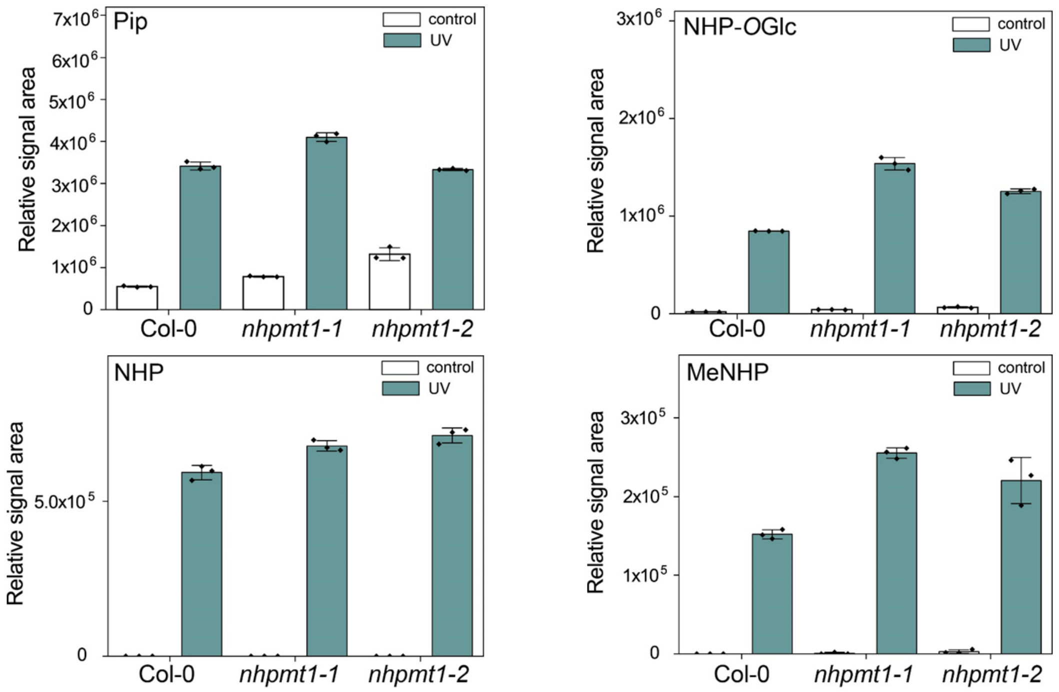

Fig. S12. Metabolite analysis of UV-treated Col-0 and *nhpmt1* mutant plants.

Col-0 and *nhpm1-1* and *nhpmt1-2* mutant plants were analyzed on their relative signal intensities of pipecolic acid (Pip), *N*-hydroxy pipecolic acid (NHP), *N*-hydroxy pipecolic acid glucoside (NHP-OGlc) and methylated NHP (MeNHP), 12 hours post UV. Metabolites were extracted using 80 % MeOH and samples were analyzed using UHPLC-MS. Mean relative signal area is shown for Pip, NHP, NHP-*O*Glc and MeNHP. Error bars indicated standard diviation. n= 3. Each replicate represents independent pools of 6-8 leaves from three plants.

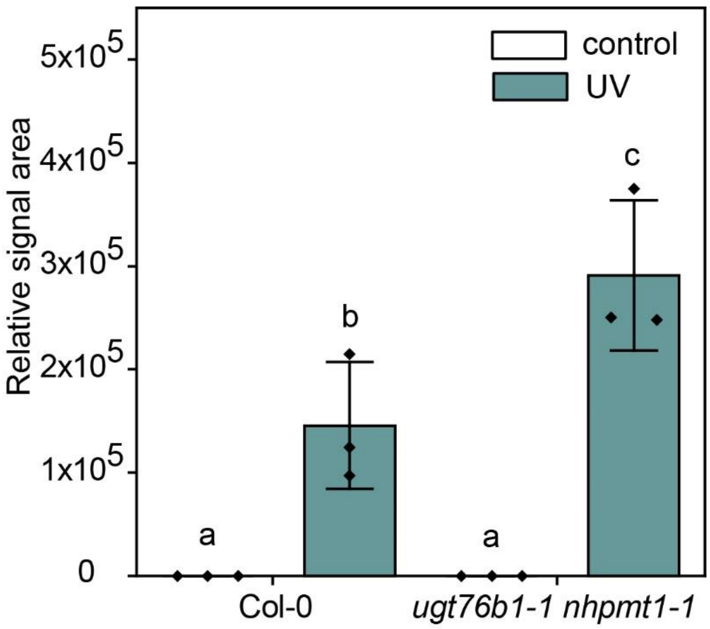

Fig. S13. MeNHP analysis of UV-stressed Col-0 vs. *ugt76b1-1 nhpmt1-1*.

Plants were treated for 20 min with UV light or kept untreated as control. Plants were incubated for 24 hours post treatment. Individual samples were collect, frozen in liquid nitrogen and retched prior to extraction. Metabolites were extracted with 80 % MeOH solution and samples were anylzed using UHPLC-MS. Mean relative signal area is shown for MeNHP. Samples represent individual pools of a total of 6-8 leaves of three plants. n=3. Letters indicate statistical differences ($p < 0.05$, one-way ANOVA post-hoc tukey-test).

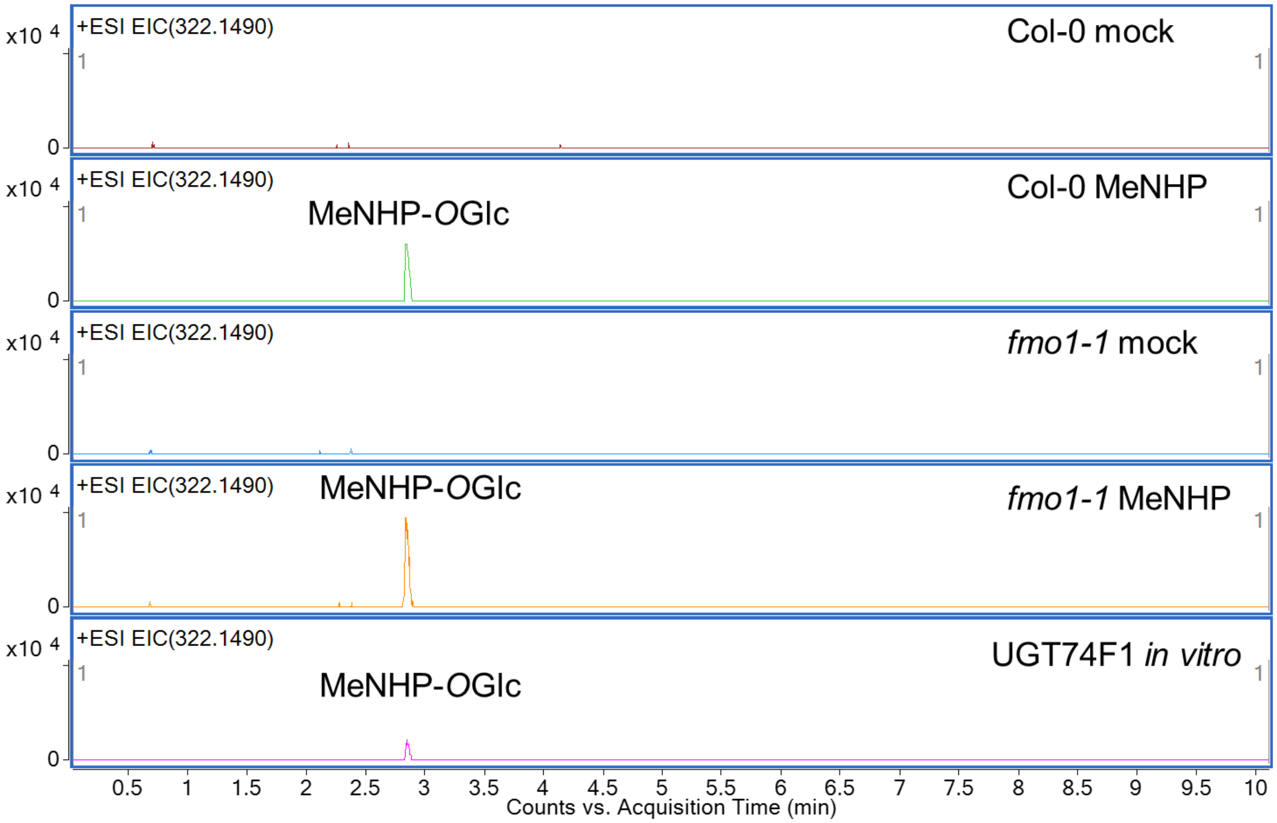

Fig. S14. Infiltration of MeNHP leads to MeNHP-*O*Glc formation, which is underlined *in vitro*.

10 mM MeNHP were infiltrated to WT and *fmo1-1* plants in 10 mM $MgCl_2$ solution. Plants were incubated for 24 hours at short day conditions. Leaves were harvested and frozen in liquid nitrogen. Metabolite extracts were analyzed by UHPLC-MS. The MeNHP-*O*Glc signal was reproduced *in vitro* with a reaction of UGT74F1, MeNHP and UDP-Glc.

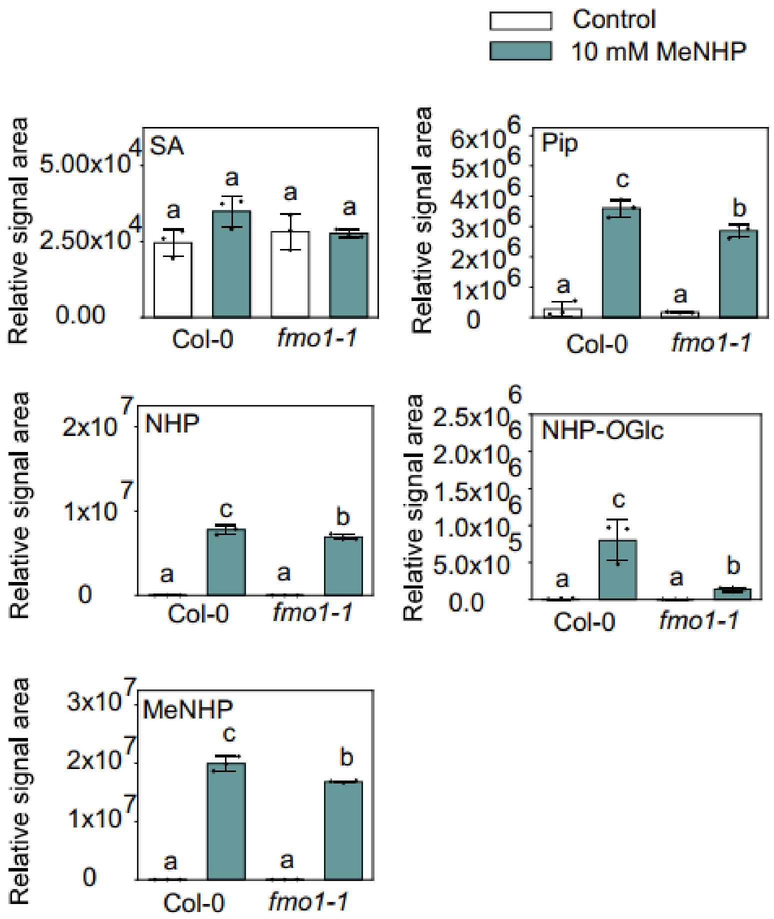

Fig. S15. Metabolite analysis after spray application of MeNHP.

10 mM MeNHP were sprayed to WT and *fmo1-1* plants in 10 mM $MgCl_2$ solution substituted with 0.1% Tween 20. Plants were let incubating for two hours in a closed environment. Afterwards, lids were removed to guarantee ideal growth conditions and the plants were incubated for another 22 hours, in long day conditions in the greenhouse. Leaves were harvested and frozen in liquid nitrogen. Metabolites were extracted using 80 % MeOH and samples were analyzed by UHPLC-MS. Mean relative signal area is shown for SA, Pip, NHP, NHP-*O*Glc and MeNHP. Error bars indicated standard diviation Letters indicate statistical differences ($p < 0.05$, one-way ANOVA post-hoc tukey-test, n=3). Each replicate represents independent pools of 6-8 leaves from three plants.

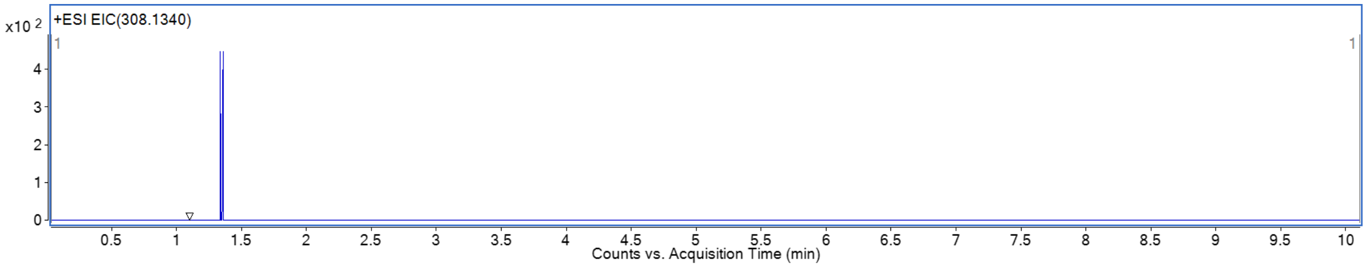

Fig. S16. UGT73D1 is not active with NHP *in vitro*.

UGT73D1 was cloned into pET28a expression vector and heterologous expressed in Bl21-expression cells. UGT73D1 was purified via ion metal affinity chromatography. Active UGT73D1 was mixed *in vitro* with 0.5 mM NHP and 0.5 mM UDP-Glc and incubated overnight. The reaction was stopped by the addition of 1/3 (*v/v*) MeOH and centrifuged prior to following UHPLC-MS analysis. The extracted ion chromatogram of $[M+H]^+$ 308.134 is shown at 0.005 mDa range.
